# Selection of aptamers against triple negative breast cancer cells using high throughput sequencing

**DOI:** 10.1038/s41598-021-87998-y

**Published:** 2021-04-21

**Authors:** Débora Ferreira, Joaquim Barbosa, Diana A. Sousa, Cátia Silva, Luís D. R. Melo, Meltem Avci-Adali, Hans P. Wendel, Ligia R. Rodrigues

**Affiliations:** 1 CEB - Centre of Biological Engineering, University of Minho, Campus de Gualtar, 4710-057 Braga, Portugal; 2 MIT-Portugal Program, Lisbon, Portugal; 3 Department of Thoracic and Cardiovascular Surgery, University Hospital Tuebingen, Calwerstr. 7/1, 72076 Tuebingen, Germany

**Keywords:** Breast cancer, Cell delivery, Diagnostic markers

## Abstract

Triple-negative breast cancer is the most aggressive subtype of invasive breast cancer with a poor prognosis and no approved targeted therapy. Hence, the identification of new and specific ligands is essential to develop novel targeted therapies. In this study, we aimed to identify new aptamers that bind to highly metastatic breast cancer MDA-MB-231 cells using the cell-SELEX technology aided by high throughput sequencing. After 8 cycles of selection, the aptamer pool was sequenced and the 25 most frequent sequences were aligned for homology within their variable core region, plotted according to their free energy and the key nucleotides possibly involved in the target binding site were analyzed. Two aptamer candidates, Apt1 and Apt2, binding specifically to the target cells with $K_{d}$ values of 44.3 ± 13.3 nM and 17.7 ± 2.7 nM, respectively, were further validated. The binding analysis clearly showed their specificity to MDA-MB-231 cells and suggested the targeting of cell surface receptors. Additionally, Apt2 revealed no toxicity in vitro and showed potential translational application due to its affinity to breast cancer tissue sections. Overall, the results suggest that Apt2 is a promising candidate to be used in triple-negative breast cancer treatment and/or diagnosis.

## Introduction

Breast cancer is the most commonly diagnosed cancer and the leading cause of death among women with nearly 2.1 million new cases diagnosed and 630,000 deaths in 2018^1^. Triple-negative breast cancer (TNBC) is a heterogeneous subtype of breast cancer, characterized by the absence of receptors for estrogen (ER), progesterone (PR), and epidermal growth factor receptor 2 (HER2) that represents approximately 15% of all breast cancer cases. TNBC patients have a worse prognosis comparing to other breast cancer subtypes due to its aggressive and metastatic nature, elevated rates of relapse, and a low response to current therapies^2–4^. Conventional chemotherapy has been the only systemic treatment option for early and advanced TNBC disease^5^. However, recently published advances have shown exciting results with poly (ADP-ribose) polymerase (PARP) inhibitors and immunotherapy agents^6–8^.

An emerging strategy in tumor-targeted therapies consists of using specific ligands, such as aptamers that are able to bind a variety of targets including proteins, small molecules, viruses, bacteria and live cells with high affinity, specificity and selectivity^9–11^. Aptamers exhibit many advantages over other ligands, such as the antibodies, since they can be easily synthesized and chemically modified, are non-toxic, and possess rapid tissue penetration and low immunogenicity^12^. Aptamers are short single-stranded deoxyribonucleic acid (ssDNA) or ribonucleic acid (RNA) oligonucleotides derived from random oligonucleotide libraries through an in vitro iterative method so-called Systematic Evolution of Ligands by EXponential Enrichment (SELEX), which involves repetitive rounds of partitioning and enrichment commonly performed with purified target proteins immobilized on a solid support^13,14^. Cell-SELEX enables the generation of aptamers directed towards cell-surface molecules by using whole living cells as targets^11,15^. It allows the screening of cell-specific aptamers without any prior knowledge of molecular signatures of the target cells, besides offering the unique ability to target specific phenotypes by performing positive–negative selection cycles to find proteins only present on the target cells^16,17^. This procedure was successfully used for selecting breast cancer targeting aptamers^18,19^. For instance, Liu et al.^16^ selected a ssDNA aptamer against MCF-7 breast cancer cells able to differentiate between breast cancer molecular subtypes.

However, the identification of new specific-target aptamers has been challenging mainly due to difficulties related to the characterization of the potential sequences from enriched libraries. Usually, the identification of enriched sequences is mainly performed by cloning and Sanger sequencing of the library resulting from several selection cycles^16,20^. Therefore, only sequenced clones are identified as possible targeting ligands, excluding candidate aptamers with good performances but with low copy numbers in the enriched library. To overcome this issue, specialized technologies have been incorporated into the original SELEX process such as high throughput sequencing (HTS) and bioinformatics analysis. HTS and bioinformatics combined with SELEX (HT-SELEX) enable the identification of a large number of aptamer candidates, total reads, frequencies of each unique sequence, distribution of each nucleotide in the random sequence and the rate of molecular enrichment^21^. Moreover, HT-SELEX allows identifying aptamers with high affinity and specificity at earlier selection rounds, which can greatly reduce over-selection which is time-consuming, and also avoid potential PCR artifacts in the amplification steps^22^.

In this study, we combined cell-SELEX with next-generation HTS and bioinformatics analysis to select ssDNA aptamer sequences that specifically bind to the metastatic TNBC cell line MDA-MB-231. The selected aptamers were further characterized and their affinity and specificity against the target cells were confirmed by flow cytometry and fluorescence microscopy. Moreover, the translational applicability of these aptamers was assessed using tumor tissue sections. Our results suggest that the selected aptamers are promising candidates for targeting and diagnosis applications in TNBC.

## Results

### Selection of aptamers against MDA-MB-231 cells through cell-SELEX

**Figure 1:**
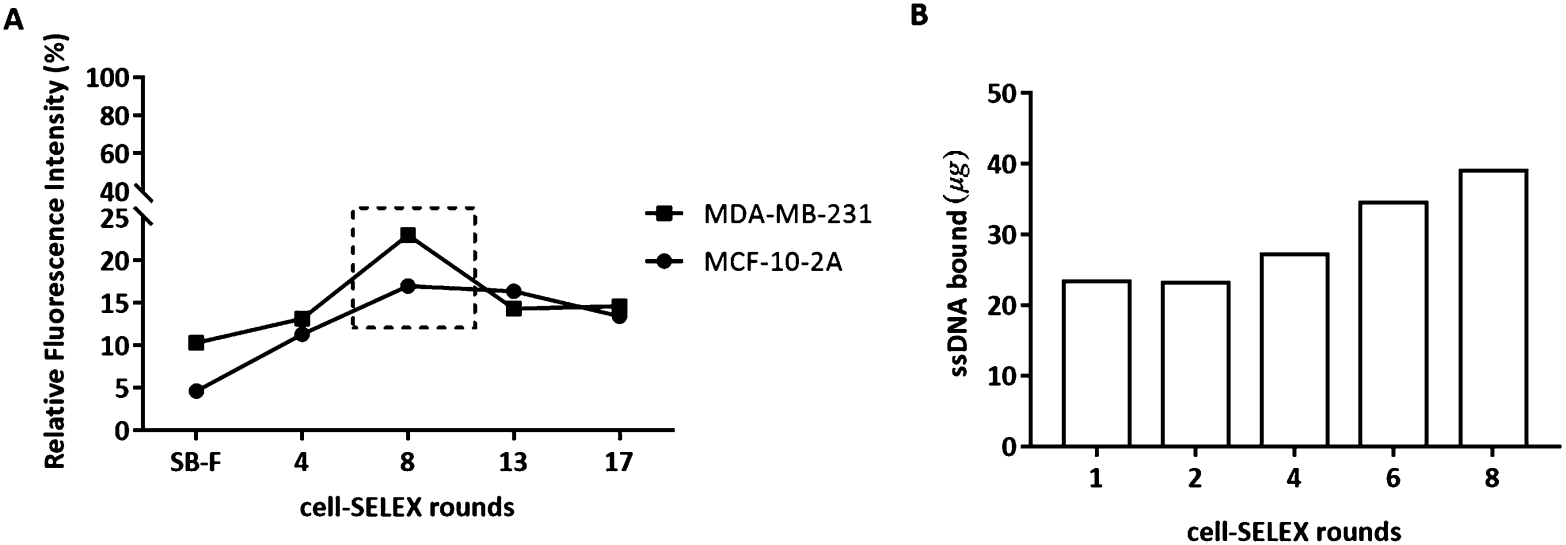
Monitoring the enrichment of the selected DNA libraries regarding the target cell line MDA-MB-231 and MCF-10-2A cells used for counter selection steps during the cell-SELEX process. (**A**) Fluorescence intensities of target and negative control cells incubated with FAM-labelled ssDNA pools from the initial library (SB-F) to the seventeenth selection cycle. (**B**) Represented is the ssDNA amount bound to the MDA-MB-231 cells from the first to the eighth selection cycle.

In the first SELEX round, a positive selection was performed. The initial ssDNA library was incubated with MDA-MB-231 cells to obtain the maximum amount of ssDNA oligonucleotides binding to the target cells. From the 2nd round onwards, the negative control cells MCF-10-2A were introduced for counter-selection to eliminate any sequences recognizing surface molecules common to both cell lines, after which the unbound sequences were again incubated with the target cells for positive selection (Table S2). Finally, the ssDNA molecules bound to the target cells were amplified by PCR to be used in the next selection round. A total of 18th rounds of selection were conducted. PCR results from each selection round were monitored by electrophoresis, showing the band for enriched ssDNA pool equal to the initial library with about 90 nucleotides (nt) (*data not shown*). Flow cytometry was used to monitor the enrichment of the ssDNA pool with binding sequences after the 4th, 5th, 8th, 13th and 17th rounds. The binding ability and specificity of the enriched ssDNA pools were evaluated by incubation with target or control cells and detection of the fluorescence signal. As shown in Fig. 1A, the fluorescence signal with target MDA-MB-231 cells was gradually increased after incubation with the enriched ssDNA pools up to the 8th round. Also, a significant difference between the fluorescence intensity for negative control and target cells after incubation with FAM-labelled ssDNA pools was observed. The results suggest that enriched sequences specific to the target cells were obtained at the 8th round, binding to approximately 20% of the cell population. A decrease of fluorescence signal for the target cells after incubation with the subsequent ssDNA pools was observed, which can be explained by the over-selection that occurs due to PCR artifacts in the amplification steps of cell-SELEX process.

The progression of the selection process was also monitored by measuring the amount of ssDNA retained on cells after washing, obtained after PCR and ssDNA generation (Fig. 1B). These results show an enrichment of ssDNA sequences binding to MDA-MB-231 cells up to the 8th round. Hence, the ssDNA pool from the 8^th^ round was further sequenced through Illumina MiSeq NGS.

### Identification of ssDNA aptamer candidates aided by bioinformatics tools

**Figure 2:**
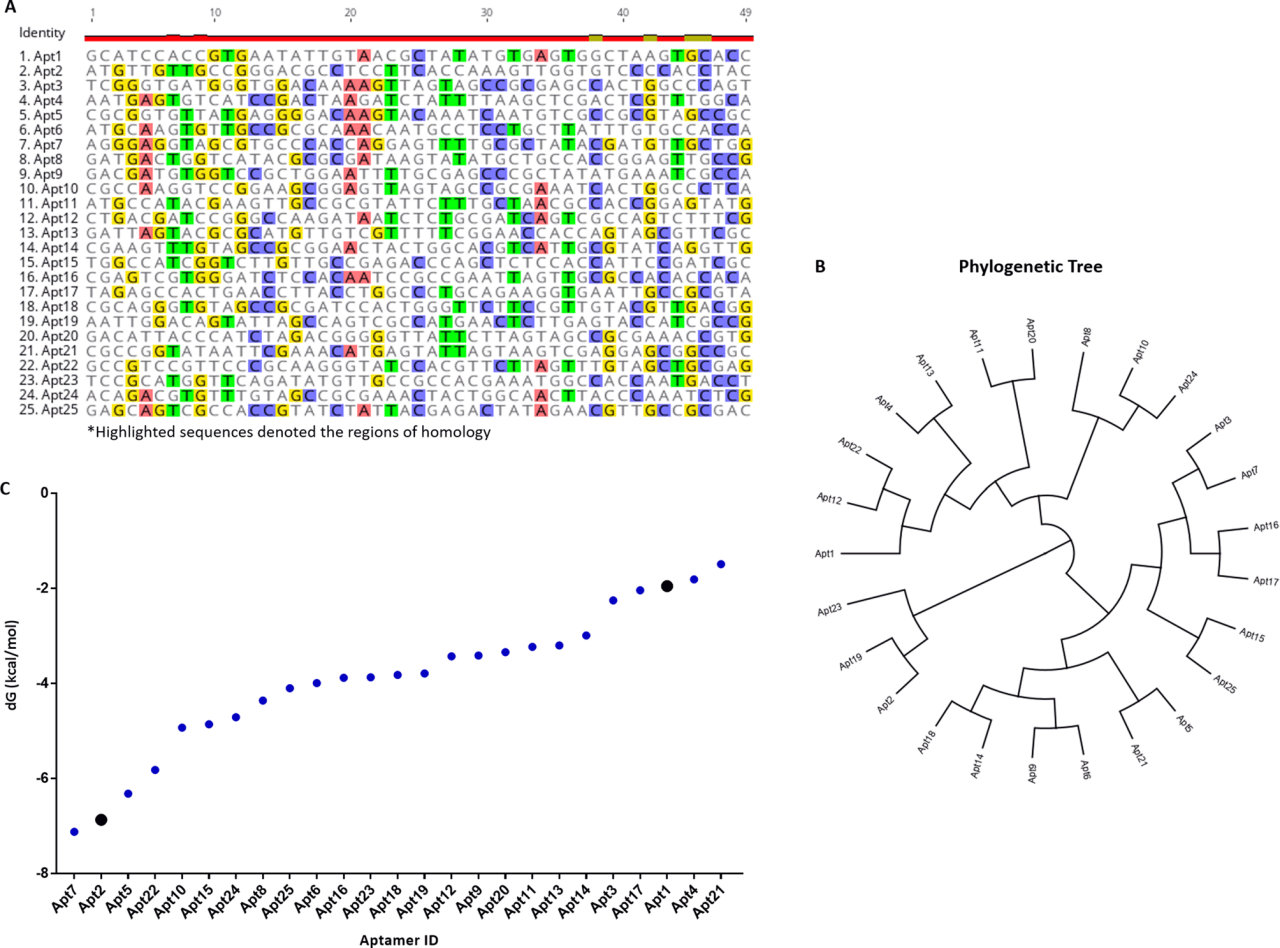
Primary structure, phylogenetic tree, and Gibbs free energy. (**A**) Multiple sequence alignments of the random region from the twenty-five top-ranked oligonucleotide sequences obtained after NGS of the eighth selection cycle. Highlighted sequences denoted the regions of homology. (**B**) Phylogenetic analysis of aptamer candidates’ sequences was performed using the Tree Builder function in Geneious software (Geneious version 9.1.4; https://www.geneious.com/). The tree distances and sequence relatedness were determined using a neighbor-joining model with no outgroup. (**C**) Gibbs free energy of the twenty-five most frequent aptamer sequences was calculated considering the flanking regions, at 37 °C, 187 mM Na^+^ and 0.5 mM Mg^2+^, using mfold web server (version 3.0, http://www.unafold.org/mfold/applications/dna-folding-form.php).

Sequencing results after an initial filtration, adapter and constant primer binding region removal and length filtration (49 nt) led to 4,699,206 total sequences, from which about 4,347,204 were unique sequences and 352,002 exhibited more than one copy in the entire sequenced pool (Table 1). The 25 most frequent oligonucleotide sequences (exhibiting only the randomized region), retrieved by either Matlab or Geneious refining, are detailed in Table 2. These sequences were further aligned for homology within their variable core region (Fig. 2A, B) and plotted according to their free energy (Fig. 2C). The analysis of the nucleotide sequences within those sequences were performed regarding sequence alignment, phylogenetic relationship, and secondary structure prediction.

**Table 1:** Next-generation sequencing (NGS) results.

Total sequences	6,513,063
Unique sequences	6,045,766
Correct sequences (49 nt)	4,699,206
Sequences (1 copy)	4,347,204
Sequences (> 1 copy)	352,002
Sequences (> 10 copies)	111
Sequences (> 20 copies)	38
Sequences (> 25 copies)	25
Sequences (> 30 copies)	17
Sequences (> 40 copies)	11
Sequences (> 50 copies)	9

Compilation of data on all the aptamer sequences obtained by NGS.

**Table 2:** Selected oligonucleotide sequence candidates. Twenty-five most frequent oligonucleotide sequences obtained after next-generation sequencing (NGS) of the 8th Cell-Selex cycle. Only the sequence corresponding to the random region is shown. Primer binding sites were neglected in this representation.

Aptamer ID	Sequences (5′–3′)	Copies
Apt1	‘GCATCCACCGTGAATATTGTAACGCTATATGTGAGTGGCTAAGTGCACC'	89
Apt2	‘ATGTTGTTGCCGGGACGCCTCCTTCACCAAAGTTGGTGTCCCCACCTAC'	86
Apt3	‘TCGGGTGATGGGTGGACAAAAGTTAGTAGCCGCGAGCCACTGGCCCAGT'	79
Apt4	‘AATGAGTGTCATCCGACTAAGATCTATTTTAAGCTCGACTCGTTTGGCA'	64
Apt5	‘CGCGGTGTTATGAGGGGACAAGTACAAATCAATGTCGCCGCGTAGCCGC'	59
Apt6	‘ATGCAAGTGTTGCCGCGCAAACAATGCCTCCTGCTTATTTGTGCCACCA'	59
Apt7	‘AGGGAGGTAGCGTGCCCACCAGGAGTTTTGCGCTATACGATGTTGCTGG'	58
Apt8	GATGACTGGTCATACGCGCGATAAGTATATGCTGCCACCGGAGTTGCCG'	56
Apt9	‘GACGATGTGGTCCGCTGGAATTTTGCGAGCCCGCTATATGAAATCGCCA'	56
Apt10	‘CGCCAAGGTCCGGAAGCGGAGTTAGTAGCCGCGAAATCACTGGCCCTCA'	49
Apt11	‘ATGCCATACGAAGTTGCCGCGTATTCTTTGCTAACGCCACCGGAGTATG'	49
Apt12	‘CTGACGATCCGGGCCAAGATAATCTCTGCGATCAGTCGCCAGTCTTTCG'	38
Apt13	‘GATTAGTACGCGCATGTTGTCGTTTTTCGGAACCACCAGTAGCGTTCGC'	37
Apt14	‘CGAAGTTTGTAGCCGCGGAACTACTGGCACGTCATTGCGTATCAGGTTG'	36
Apt15	‘TGGCCATCGGTCTTGTTGCCGAGACCCAGCTCTCCACCATTCCGATCGC'	35
Apt16	‘CGAGTCGTGGGATCTCCACAATCCGCCGAATTAGTTGCGCCACACCACA'	34
Apt17	‘TAGAGCCACTGAACCTTACCTGGCCCTGCAGAAGGTGAATTGCCGCGTA'	31
Apt18	‘CGCAGGGTGTAGCCGCGATCCACTGGGTTCTTCCGTTGTACGTTGACGG'	29
Apt19	‘AATTGGACAGTATTAGCCAGTCGCCATGAACTCTTGAGTACCATCGCCG'	29
Apt20	‘GACATTACCCATCTAGACGGGGGTTATTCTTAGTAGCCGCGAAACCGTG'	29
Apt21	‘CGCCGGTATAATTCGAAACATGAGTATTAGTAAGTCGAGGAGCGGCCGC'	28
Apt22	‘GCCGTCCGTTCCCGCAAGGGTATCCACGTTCTTAGTTTGTAGCTGCGAG'	28
Apt23	‘TCCGCATGGTTCAGATATGTTGCCGCCACGAAATGGCCACCAATGACCT'	28
Apt24	‘ACAGACGTGTTTGTAGCCGCGAAACTACTGGCAACTTACCCAAATCTCG'	25
Apt25	‘GAGCAGTCGCCACCGTATCTATTACGAGACTATAGAACGTTGCCGCGAC'	25

The aligned N49 regions showed some conserved sequences such as ‘GTTG’ (Apt2, 6, 7, 8, 11, 13–16, 18, 23 and 25); ‘X_1_GCTGCX_2_’ where X_1_ can be either T or A and X_2_ can be G or C (Apt8 and 22); ‘TGGTX’ where X can be either C, T or G (Apt2, 8, 9 and 23); ‘X_1_TTGCCGX_2_’ where X_1_ can be G or A and X_2_ can be G, C or A (Apt2, 6, 8, 11, 15, 17, 23, 25); ‘XCAAGTX’ where X can be both either G or A (Apt5 and 6); ‘XGAGT’ where X can be T, C or G (Apt1, 4, 7, 8, 10, 11, 16, 19 and 21); ‘AAGT’ (Apt1-3, 5, 6, 8, 11, 14 and 21); ‘X_1_GGAGX_2_’ where X_1_ can be G, A or C and X_2_ can be G, T or C (Apt7, 8, 10, 11 and 21); and ‘GCCG’ (Apt2, 3, 5, 6, 8, 10, 11 and 14–25) (Fig. 2A). Furthermore, the selected aptamer sequences showed a distinct evolutionary tree (Fig. 2B), where three main families of related aptamers were identified, meaning that they do not have a similar sequence for conserved nucleotides. Apt1, Apt12, Apt22, Apt4, Apt13, Apt11, Apt20, Apt8, Apt10 and Apt24 belong to one family; Apt3, Apt7, Apt16, Apt17, Apt15, Apt25, Apt21, Apt6, Apt9, Apt 14 and Apt18 to another; and Apt2, Apt19 and Apt23 to the last one. According to their free energy, Apt7 and Apt2 have the lowest free energy and Apt4 and Apt21 the highest ones (Fig. 2C). The lower the free energy, the more stable the secondary structure.

**Figure 3:**
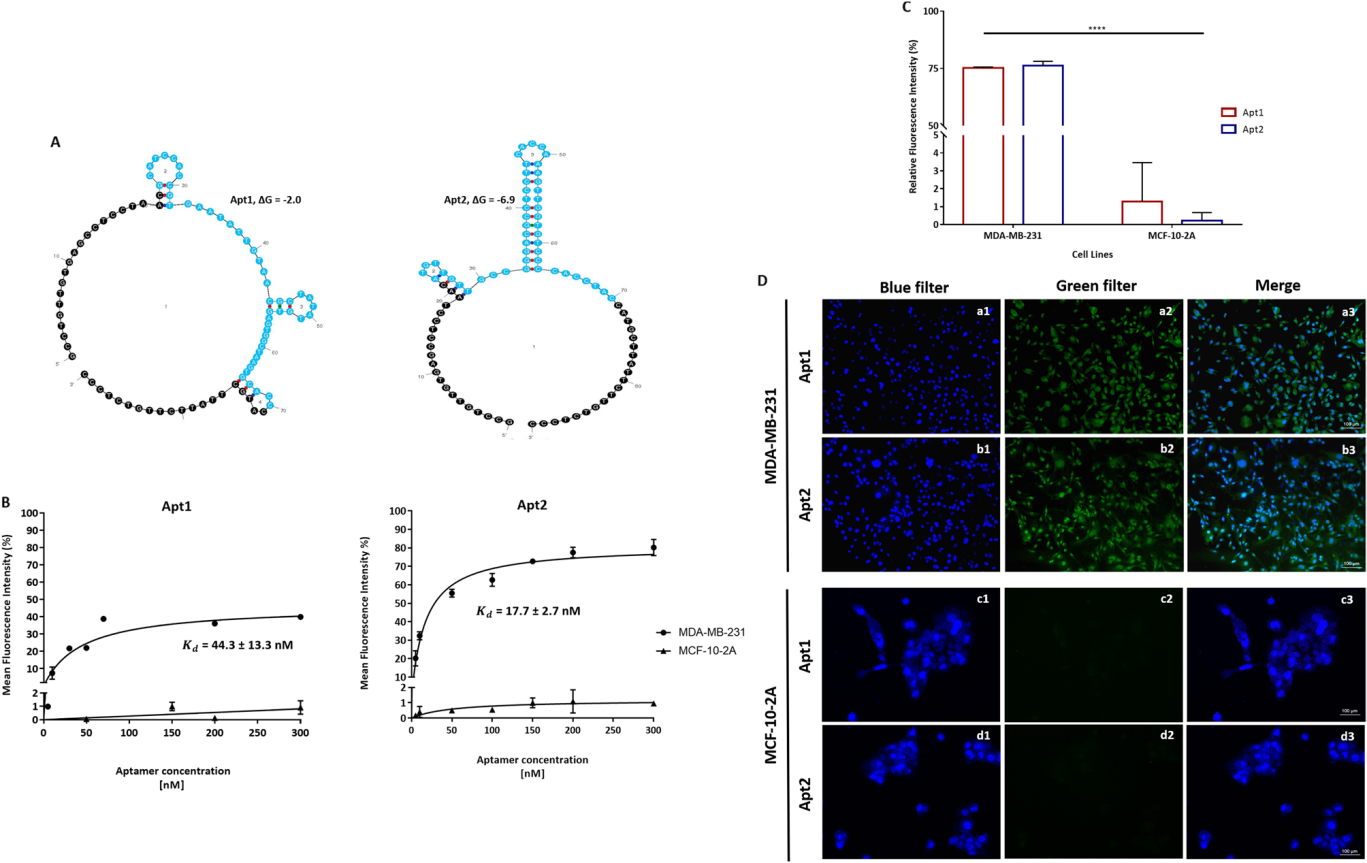
Secondary structure, equilibrium dissociation constant determination and binding ability of the selected aptamers. (**A**) Predicted secondary structures for two aptamer candidates, Apt1 and Apt2, selected for further in vitro experiments. The presented predicted secondary structures were the ones with lowest ΔG, i.e. the highest stability using the temperature 37 °C, 187 mM Na^+^, and 0.5 mM Mg^2+^, as calculated using the mfold web server (version 3.0, http://www.unafold.org/mfold/applications/dna-folding-form.php). Constant sequence regions are highlighted in black, and blue represents the random regions. (**B**) Binding curve of aptamers Apt1 and Apt2 with MDA-MB-231 and MCF-10-2A cells. The cells were incubated with increasing concentrations of FAM-labelled aptamers and assessed by flow cytometry. Equilibrium dissociation constants $\left( {K_{d} } \right)$ (nM) were calculated using GraphPad Prism 7, under the non-linear fit model, one-site non-competitive binding to fluorescent population ratio at used aptamer concentrations. (**C**) Binding ability of FAM-labelled aptamers, Apt1 and Apt2, with MDA-MB-231 and MCF-10-2A cells at 37 °C assessed by flow cytometry. All data are expressed as the mean ± SD of three independent experiments. Two-way ANOVA indicates statistically significant differences within the group assessed by Sidak’s post-test and denoted as follows: ****ρ ≤ 0.0001. (**D**) Microscopy results expressing the fluorescence imaging of MDA-MB-231 (a and b) and MCF-10-2A (c and d) cells incubated with FAM-labelled Apt1 and Apt2 at 37 °C, respectively. (1) blue filter, nuclei stained with DAPI, (2) green filter, Apt1 or Apt2, and (3) merge of all filters (1 and 2).

Secondary structures of the 25 selected aptamer sequences were predicted using the Mfold web server (version 3.0, http://www.unafold.org/mfold/applications/dna-folding-form.php)^23^ to find their folding characteristics considering the primer and random regions (Fig. 3A and Table S3). For instance, Apt5, 6, and 21 show one stem loop (SL) structure, whereas Apt2, 3, 7, 8, 10, 12, 14–18, 22, and 24 show two. Moreover, Apt1, 4, 6, 13, 20, 23, and 25 possess three SL structures, while Apt11 and Apt19 have four. Additionally, the nucleotides involved in the formation of the SL structures were investigated. The conserved sequence ‘GTTG’ is found in the SL region of Apt2, 6, 7, 13, 15, 16, 18, 25, thus meaning that these aptamers are able to recognize the same target, despite exhibiting overall a low homology and conservation with each other. Moreover, the motif ‘TTGCCG’ is found in a SL structure of Apt15 and 25; ‘CAAGT’ in Apt5 and 6; ‘GAGT’ in Apt7, 8 and 21; ‘AAGT’ in Apt 5, 6, 11, 14, 21; ‘GGAG’ in Apt7 and 8; and ‘GCCG’ in Apt14-16, 19, 22 and 25.

Among the 25 selected aptamer sequences, two candidate aptamers (Apt1 and Apt2) were chosen based on their pool repeatability, as well as on their different conserved sequences and secondary structures. Both aptamers were synthesized with FAM labels to be used in the following characterization experiments.

### Affinity properties of the of Apt1 and Apt2 against MDA-MB-231 cells

In order to evaluate the binding affinity of the aptamer candidates Apt1 and Apt2, their $K_{d}$ values were calculated using Eq. (1). Figure 3B shows the aptamers binding saturation curves with the target MDA-MB-231 cell lines, leading to $K_{d}$ values of 44.3 ± 13.3 nM and 17.7 ± 2.7 nM, respectively. Saturation curves with the negative control MCF-10-2A cell line for both aptamers are also shown.

To evaluate the ability of both aptamers to bind to MDA-MB-231 (target) and MCF-10-2A (control) cells, fluorescence microscopy imaging and flow cytometry were performed (Fig. 3C, D). The microscopy and cytometry results are in very good agreement. Apt1 and Apt2 bound to about 75.2 ± 0.4% and 76.2 ± 1.9% of the overall MDA-MB-231 cell population, respectively. Furthermore, it is also shown that for MCF-10-2A, almost no positive signal was detected (1.3 ± 2.2% to Apt1 and 0.2 ± 0.5% to Apt2), thus confirming the specificity of both aptamers. The microscopy results clearly show a bright fluorescence signal for the MDA-MB-231 cell population after incubation with the aptamers, being more pronounced for Apt2. As expected, no fluorescence signal was observed for the control cells, thus corroborating the cytometry results.

### Apt1 and Apt2 specifically target cell surface receptors

**Figure 4:**
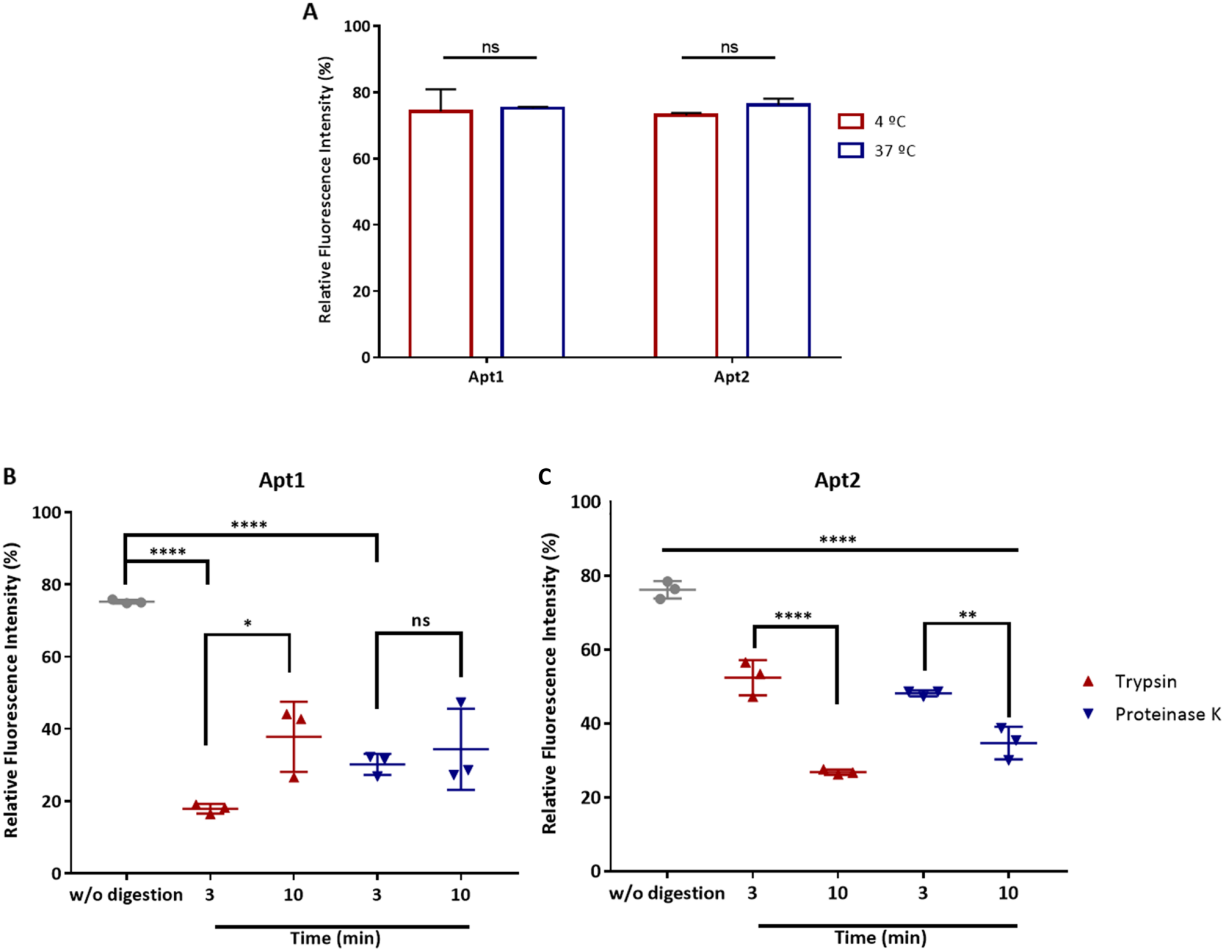
Binding characterization of Apt1 and Apt2 aptamers to MDA-MB-231 cells. (**A**) Effect of incubation temperature on the binding ability of Apt1 and Apt2 in MDA-MB-231 cells. The cells were incubated with FAM-labeled Apt1 and Apt2 at 4 °C or 37 °C and analyzed by flow cytometry. (**B**) Binding ability of aptamers Apt1 and Apt2 with MDA-MB-231 cells treated with trypsin or proteinase K. The cells were treated with trypsin or proteinase K at 37 °C for 3 or 10 min and then incubated with FAM-labelled Apt1 or Apt2, respectively. The binding ability of FAM-labelled Apt1 and Apt2 was analyzed by flow cytometry. All data are expressed as the mean ± SD of three independent experiments. One-way ANOVA indicates statistically significant differences within the group assessed by Tukey post-test and denoted as follows: ****ρ ≤ 0.0001, **ρ ≤ 0.01, *ρ ≤ 0.1 and ns ρ > 0.05.

To investigate whether the incubation temperature could affect the binding ability of both aptamers, MDA-MB-231 cells were incubated with them at different temperatures and then analyzed by flow cytometry. MDA-MB-231 cells showed similar fluorescence intensity patterns after incubation with both aptamers at both temperatures (4 °C and 37 °C), hence demonstrating that the incubation temperature has almost no effect (*p* > 0.05) on the binding capacity of the selected aptamers (Fig. 4A). In addition, MDA-MB-231 cells, pretreated with trypsin or proteinase K for 3 and 10 min, were incubated with the FAM-labelled aptamers, and were further analyzed by flow cytometry. Both aptamers lost the binding ability to their target cells after treatment with either proteinase K or trypsin at both exposure times (Fig. 4B).

**Figure 5:**
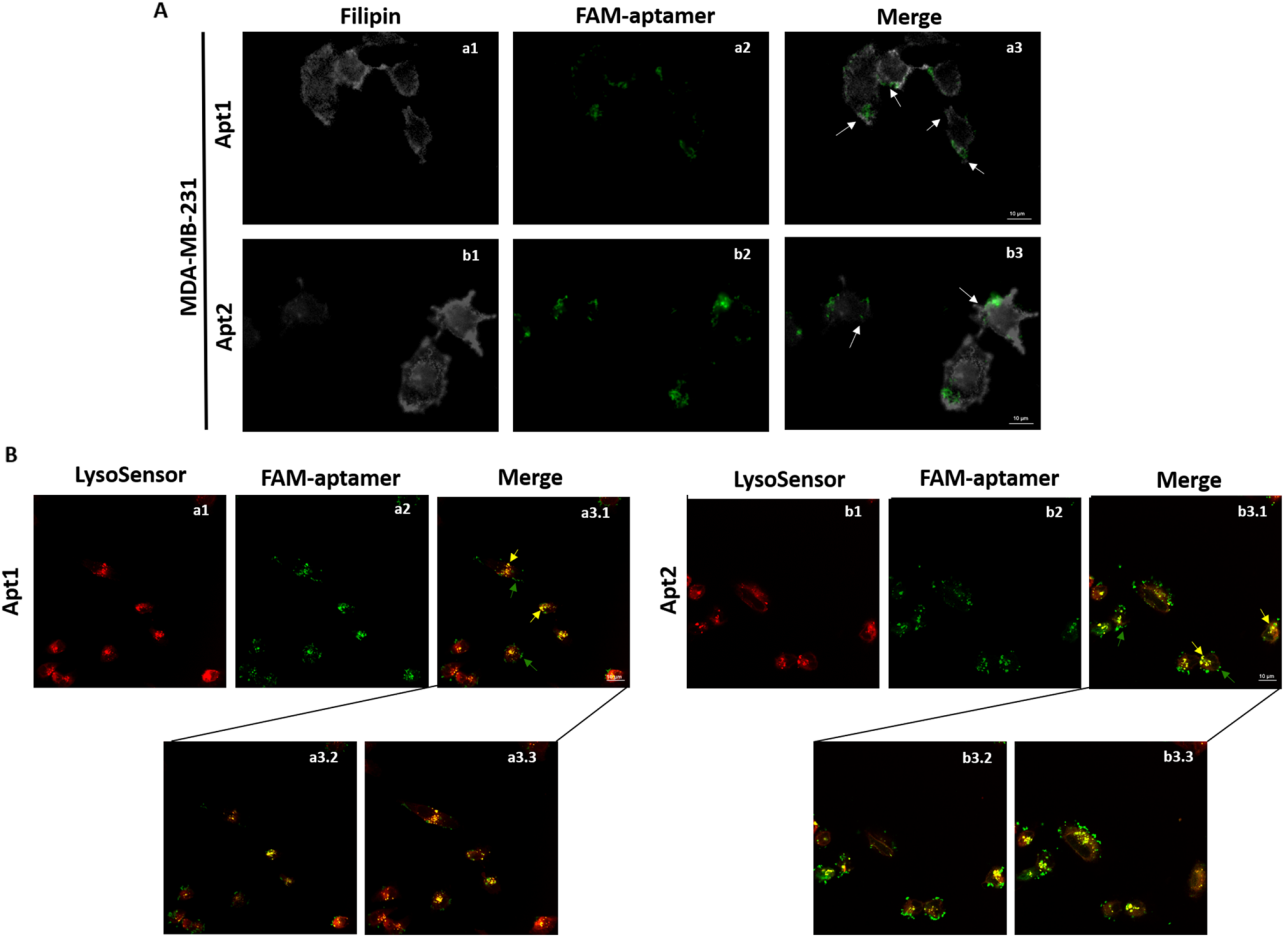
Co-localization of Apt1 and Apt2 with MDA-MB-231 cells. FAM-labelled Apt1 and Apt 2 were incubated at 37 °C with MDA-MB-231 cells and then with filipin (**A**) and lysoSensor Red DND-99 (**B**). (**A**) Fluorescence imaging of MDA-MB-231 cells stained with filipin (1) and incubated with FAM-labelled (2) Apt1 (a) and Apt2 (b). White arrows point out membrane staining overlay with aptamer binding (3). (**B**) Confocal images of MDA-MB-231 cells labelled with lysoSensor (1) to track lysosomes location and incubated with FAM-labelled (2) Apt1 (a) and Apt2 (b) at 37 °C. Merged files are representative of co-localization of Apt1 and Apt2 with lysosensor (3.1). Images were obtained under confocal scanning laser microscope using a 60X oil immersion objective. Green arrows point out membrane location of FAM-labelled Apt and yellow ones the co-localization of FAM-labelled Apt with lysoSensor. Insert: co-localization of FAM-labelled Apt1 and Apt2 with lysoSensor observed in 2 optical sections of MDA-MB-231 cells (3.2 and 3.3).

A co-localization experiment was also performed using the naturally fluorescent filipin and both labelled aptamers (Fig. 5A). Apt1 and Apt2 showed a notorious binding to the MDA-MB-231 cell membrane, corroborating the cytometry results. In addition, to delineate cell cytoskeleton, MDA-MB-231 cells were stained with CF 568 Phalloidin and an observable green fluorescence (at a less extent than the observed with filipin due to the great amount of washes during the protocol) was also observed at the membrane level (Fig. S1). In the end, to trace the potential cellular uptake of both aptamers, cells were incubated with lysoSensor (Fig. 5B). Again, a membrane staining was clearly observed for both aptamers (pointed out by the green arrows) but, interestingly, it was also found some aptamer uptake, represented by the yellow dots (highlighted by the yellow arrows).

### Apt1 and Apt2 are MDA-MB-231 cell-specific

**Figure 6:**
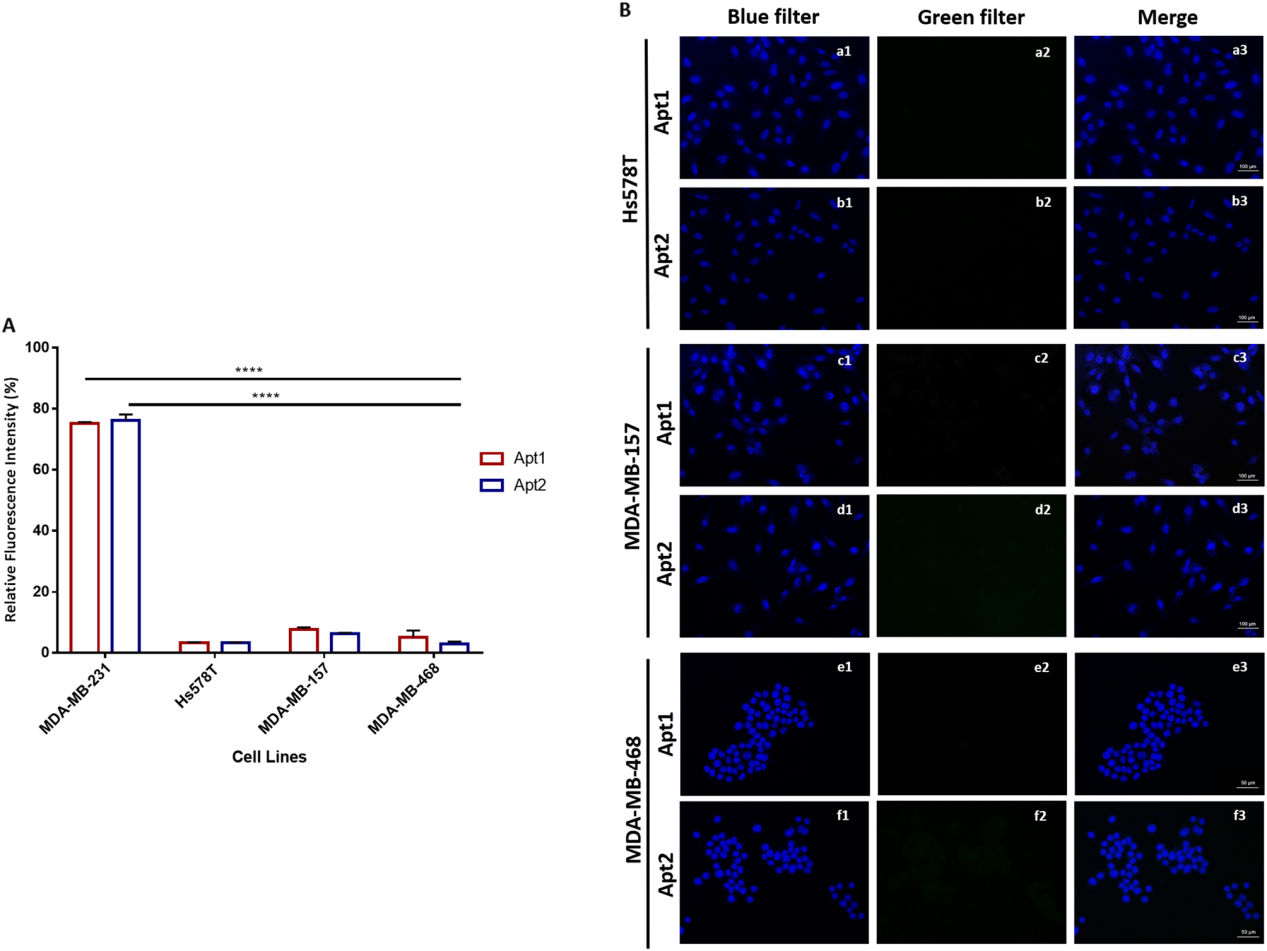
Binding specificity of Apt1 and Apt2 aptamers to other TNBC cell lines. (**A**) FAM-labelled Apt1 and Apt2 were incubated at 37 °C with Hs 578T, MDA-MB-157 and MDA-MB-468 cell lines and analyzed by flow cytometry. Two-way ANOVA indicates statistically significant differences within the group assessed by Sidak’s post-test and denoted as follows: ****ρ ≤ 0.0001, ***ρ ≤ 0.001 and **ρ ≤ 0.001. (**B**) Microscopy results showing the fluorescence imaging of Hs 578T (a and b), MDA-MB-157 (c and d) and MDA-MB-468 (e and f) cells incubated with FAM-labelled Apt1 and Apt2 at 37 °C, respectively. (1) blue filter, nuclei stained with DAPI, (2) green filter, Apt1 or Apt2, and (3) merge of all filters (1 and 2).

Binding assays against three additional TNBC cells (Hs 578T, MDA-MB-157 and MDA-MB-468) were also performed (Fig. 6). The binding specificity was evaluated by flow cytometry and microscopy. The aptamer binding affinity was significantly lower for Hs 578T (ρ ≤ 0.0001), MDA-MB-157 (ρ ≤ 0.0001) and MDA-MB-468 (ρ ≤ 0.0001) when compared to MDA-MB-231 cells. About 3.3 ± 0.2% and 3.3 ± 0.1%, 7.7 ± 0.7% and 6.3 ± 0.2%, 5.1 ± 2.2% and 2.9 ± 0.8% of the overall Hs 578T, MDA-MB-157 and MDA-MB-468 cell population was bound to the Apt1 and Apt2, respectively (Fig. 6A). Under microscopy, no detectable fluorescence was observed for TNBC cell lines in study (Fig. 6B). Moreover, the aptamer binding specificity was assessed for other two breast cancer cell lines, including MCF-7 (ER^+^, PR^+^, HER2^-^) and MDA-MB-453 (ER^-^, PR^-^, HER2^+^) by flow cytometry and microscopy (Fig. S2). As observed in Fig. S2A, the binding signal was significantly decreased for MCF-7 (ρ ≤ 0.0001) and MDA-MB-453 (ρ ≤ 0.0001) as compared to the target MDA-MB-231 cells, with 9.0 ± 0.02% and 7.9 ± 5.2%, 2.0 ± 0.9% and 0.9 ± 1.1% of the overall population bound to Apt1 and Apt2, respectively. No measurable fluorescence was detected under microscopy (Fig. S2B).

### Apt2 could be used as a targeting moiety in targeted therapies

**Figure 7:**
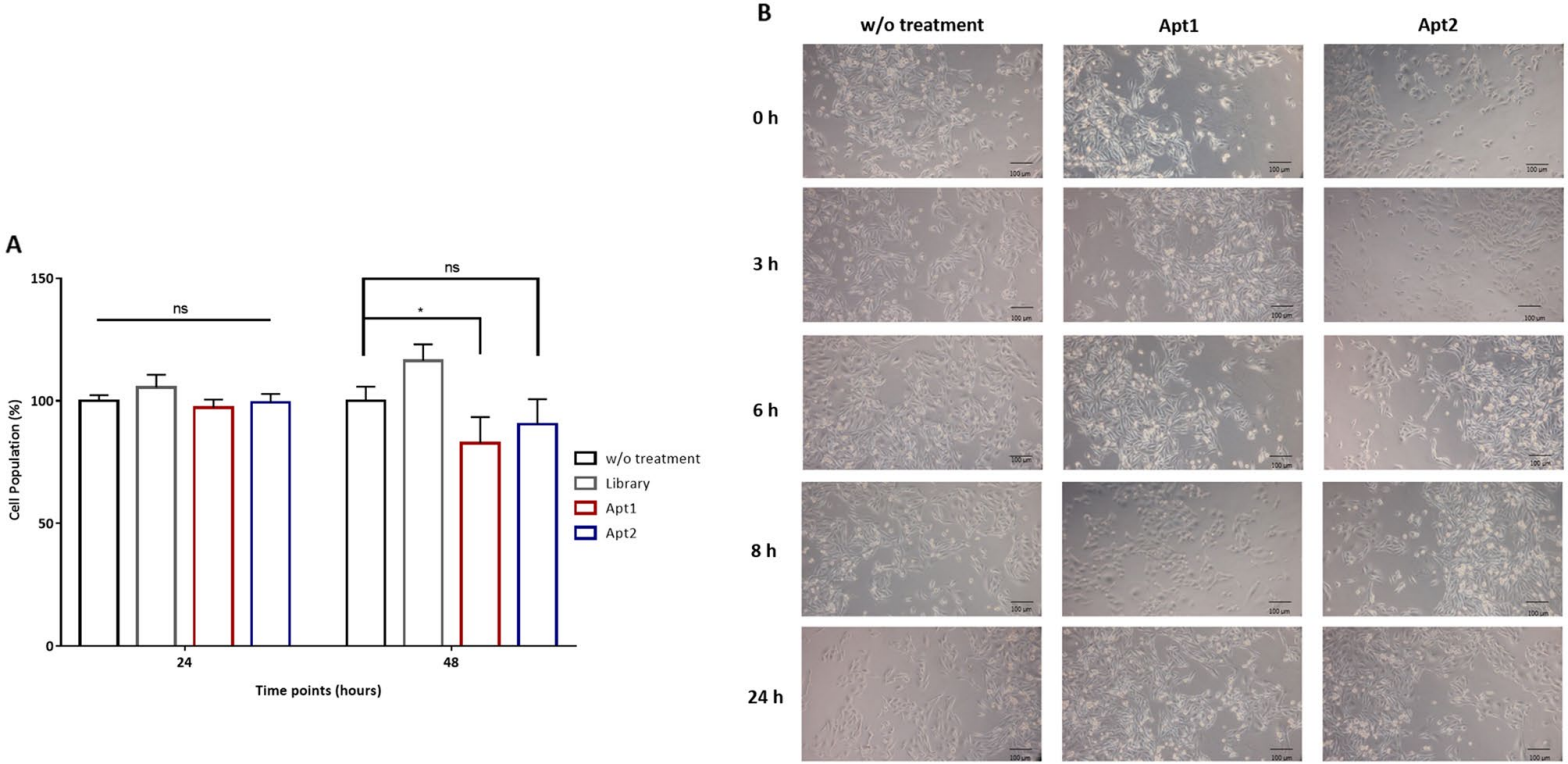
In vitro toxicity of Apt1 and Apt2. (**A**) Cell viability was evaluated by MTT assay. MDA-MB-231 cells were incubated with Apt1 and Apt2, respectively, for 24 and 48 h. The ssDNA library was used as a negative control. All data are expressed as the mean ± SD of three independent experiments. Two-way ANOVA indicates statistically significant differences within the group assessed by Sidak’s post-test and denoted as follows: *ρ ≤ 0.1 and ns ρ > 0.05. (**B**) Microscopy images of MDA-MB-231 cells treated with Apt1 or Apt2 for 0 h, 3 h, 6 h, 8 h, and 24 h. Non-treated cells were used as negative controls.

The effects of the selected aptamers on MDA-MB-231 cells viability were evaluated at two-time intervals (24 and 48 h). Cell viability was assessed using the standard colorimetric MTT assay. MDA-MB-231 untreated cells were used as the positive control and normalized to 100% cell viability. As illustrated in Fig. 7, after 24 h of incubation with 250 nM of both aptamers, no significant survival rate change was found, suggesting that both are non-toxic. The same trend was found for Apt2 for a 48 h incubation. However, for Apt1, a significant decrease in cell viability (ρ ≤ 0.05) was observed at this exposure time. The morphological changes resulting from incubation of cells with both aptamers at different time points up to 24 h (non-toxic exposure time for both aptamers) were also studied. As shown in Fig. 7B, no observable differences between the different conditions were found for both aptamers.

### Apt2 has the potential for translational application to clinical oncology

**Figure 8:**
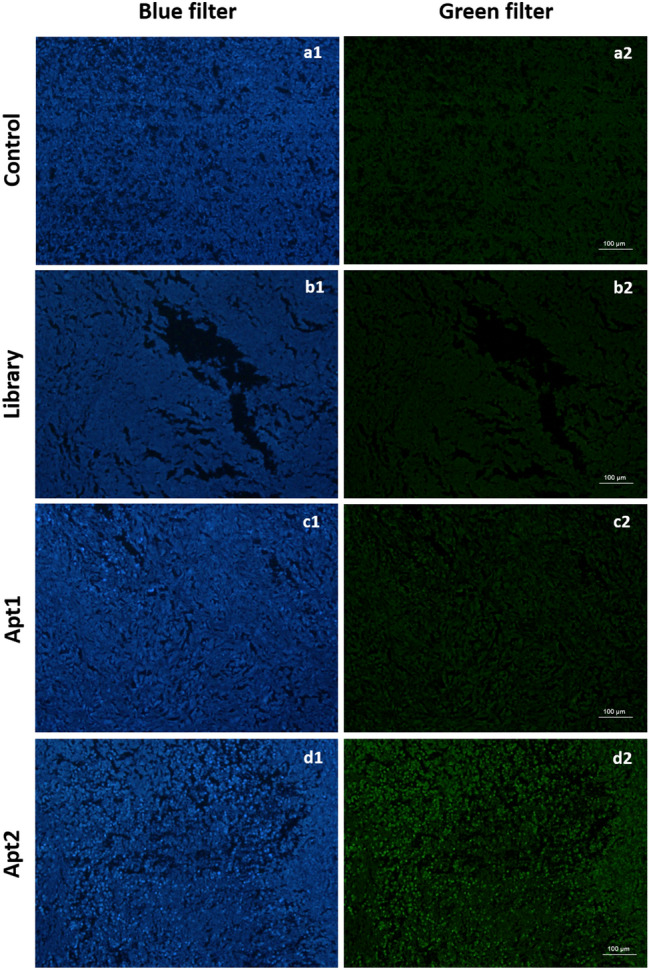
In vitro binding ability of Apt1 and Apt2 to human breast cancer tissue section detected through immunofluorescence staining. Control stands for experiments performed without FAM-labelled aptamer (**a**); with FAM-labelled ssDNA library (**b**) and with FAM-labelled Apt1 (**c**) and Apt2 (**d**), respectively. (1) blue filter, nuclei stained with DAPI and (2) green filter, FAM-labelled library, Apt1, or Apt2.

Besides the selected aptamer's ability to recognize MDA-MB-231 cells, we further evaluated their ability to target breast cancer tissues (Fig. 8). For that purpose, fluorescence microscopy was used to image breast tissue sections after incubation with both FAM-labelled aptamers and the library. A visible green fluorescence signal of FAM-labelled Apt2 was detected in human breast tissues and no visible signal was found either for Apt1 or library.

## Discussion

Breast cancer is the worldwide leading cause of deaths among women, being TNBC the most aggressive subtype with no approved targeted therapy. The identification of new molecules such as aptamers able to specifically target breast cancer may lead to the development of novel targeted therapies. Herein, cell-SELEX was used to select specific aptamers against the human breast cancer cell line MDA-MB-231, which is a good TNBC model due to its aggressive and metastatic nature.

ssDNA aptamers were identified by cell-SELEX against MDA-MB-231 cells and using the normal breast cell line MCF-10-2A for counter-selection to exclude all aptamers that bind in a non-specific way to cancer cells. The enrichment of the ssDNA pool with binding sequences was monitored, being the ssDNA pool from the 8^th^ round further sequenced by NGS. Afterwards, the sequencing results were filtered and the 25 most frequent oligonucleotide sequences were analysed regarding the sequence alignment and phylogenetic relationship. This type of analysis can provide useful insights on the evolutionary process and on key nucleotides that may be involved in the target binding site^24^. The selected aptamer sequences showed a distinct evolutionary tree, where three main families of related aptamers were identified, meaning that they do not have a similar sequence for conserved nucleotides.

Based on this analysis, it is premature to affirm that these conserved sequences are part of the target binding without confirming whether these regions of interest can contribute to form stems, loops or bulges. All aptamer sequences exhibit a predicted folded-secondary structure with a degree of SL structures. From those, two candidate aptamer sequences, Apt1 and Apt2, were chosen based on the sequences’ repeatability on the entire pool, but also on their different conserved sequences and secondary structures.

A growing number of delivery strategies using cell-specific aptamers as targeting ligands have been proposed^25,26^, given their ability to distinguish small differences in the cell surface protein signature of heterogeneous cancer cells. Therefore, to determine the binding affinity of Apt1 and Apt2 towards MDA-MB-231 cells, their $K_{d}$ values were assessed, giving values of 44.3 ± 13.3 nM and 17.7 ± 2.7 nM, respectively. Various cell-SELEX protocols have been developed to isolate cell-specific aptamers. Li et al.^16^ selected the aptamer LXL-1 ($K_{d}$ = 44 ± 8 nM) against MDA-MB-231 cells. More recently, another approach to select aptamers against circulating tumor MDA-MB-231 cells was attempted. A panel of 5 specific aptamers were identified, from which the M3 aptamer displayed the highest affinity with a $K_{d}$ of 45.6 ± 1.2 nM^19^.

In both abovementioned reports, MDA-MB-231 cell-specific aptamers were identified after Sanger sequencing. NGS revolutionized the aptamer field by increasing the number of reads from few (Sanger sequencing) to millions (HTS methods), thus increasing the probability of finding the best ligands for a given target. The $K_{d}$ values of the aptamers identified in our work are comparable, in the case of Apt1, or with a higher affinity to the target (about 2.5-fold higher), demonstrated for Apt2. A possible explanation is the use of NGS that, as mentioned, enables the retrieval of aptamers with better affinity to the target cells. Even though, this assessment is not straightforward as aptamers were selected using different experimental setups, including different libraries and protocol conditions, making it difficult to do a side by side comparison.

Aptamer selection at other temperatures rather than 37 °C, for instance, 4 °C, may result in poor binding ability at the physiological temperature^27^. However, ssDNA could be non-specifically taken up at physiological temperatures by cells through a complex process called endocytosis that occurs via receptor-mediated pathways (clathrin-mediated, caveolae-mediated, macropinocytosis and clathrin and caveolae-independent pathways)^28^. Therefore, MDA-MB-231 cells were incubated with both aptamers at different temperatures, showing similar fluorescence intensity patterns.

To preliminary assess whether putative targets of these aptamers are membrane proteins on the cell surface, MDA-MB-231 cells were pre-treated with either trypsin or proteinase K, where both aptamers showed loss of binding ability. These results suggest that the target of both aptamers is associated with epitopes on the cell surface proteins, since proteinase K and trypsin are able to digest extracellular domains of the cell surface without disrupting other components present in the cytosol. Moreover, incubation with filipin showed a clear co-localization with Apt1 and Apt2 at the membrane. Filipin is a naturally fluorescent polyene antibiotic that binds to cholesterol, and because of that, has been widely used as a probe for sterol location in biological membranes^29^.

In addition, to determine if both aptamers have the ability to be internalized upon target membrane binding, MDA-MB-231 cells were incubated with Apt1 or Apt2 and stained with lysoSensor, for fluorescent staining of acidic organelles as lysosomes. A strong accumulation of aptamer on the cell outer margins (pointed out by the green arrows) was again observed, corroborating the abovementioned results. Interestingly, a yellowish signal (highlighted by the yellow arrows) originated inside the cells and co-localized with lysoSensor was also observed. The results suggest that the target molecule of Apt1 and Apt2 is very likely a membrane protein and can be further internalized into cells.

Other authors also identified aptamers that are able to target membrane proteins and afterward are internalized. TLS11a is an aptamer generated by cell-SELEX that exhibit a great affinity against the liver cancer cells^30,31^. Meng et al.^30^ demonstrated the internalization ability of TLS11 upon targeting the cell membrane of LH86 cells. These authors generated a selective TLS11a aptamer-doxorubicin (DOX) complex to deliver DOX to liver cancer cells. Based on our results, the aptamer’s ability to target membrane receptors and subsequent internalization upon binding, makes them interesting for the development of targeted drug delivery systems and/or specific diagnosis methods. Moreover, it also proves the clear potential of cell-SELEX to generate specific ligands to target molecules in types of cancer that offer limited treatment options. Nevertheless, identifying the receptor(s) for these aptamers remains an open question worthy of further investigation.

As previously described, none of the selected aptamers were able to bind the non-tumorigenic epithelial human breast cell line MCF-10-2A, suggesting their specific binding to the target MDA-MB-231 cells. However, it was not clear if these aptamers could bind or recognize other TNBC cell lines, as Hs 578T, MDA-MB-157 and MDA-MB-468. No detectable fluorescence was observed, implying that the selected aptamers recognize cell surface receptors only present in MDA-MB-231 cells, but absent in the other cell lines assayed. Experiments with non-TNBC cell lines, as MCF-7 and MDA-MB-453, were also conducted to infer specificity and again no fluorescence was detected. MDA-MB-231, Hs 578T and MDA-MB-157 are highly invasive cell lines that have been grouped as basal B by Neve et al.^32^ or even characterized as mesenchymal-like by Lehmann et al. in a later study^33^. Contrarily, MDA-MB-468 was grouped by both research teams as basal A. Finding such aptamers able to stratify such similar TNBC cell lines could be very promising, besides providing insights about novel therapeutic cell-surface targets.

Aptamers need different times to interact with their targets and to produce a cellular response^34^. Furthermore, toxicity is a key limiting factor in the clinical translation and applicability of aptamers. Cell viability was assessed using a standard colimetric experiment that showed that for Apt2 no significant survival rate change was found, independently of the time assayed. However, for Apt1 a significant decrease in cell viability was observed at time point 48 h. The mechanisms underlying the cell viability decrease mediated by Apt1 are not fully understood. Based on these results, we foresee that Apt2 is stable enough for subsequent in vivo studies and possibly will also be useful for future clinical applications.

Aptamers can be used as therapeutic moieties covalently or non-covalently conjugated with a drug to form aptamer-drug conjugates to increase the therapeutic response on target cells^35^. In addition, aptamers can be conjugated to nanoparticles such as liposomes, micelles, polymeric nanoparticles and quantum dots to carry anti-cancer drugs, and to guide the therapeutic reagents to the target cells^35^. Hu et al.^36^ exploited the targeting ligand Mucin 1 (MUC1) aptamer for carrying DOX to cancer cells. DOX was intercalated into the DNA aptamer MA3, which binds to MUC1, to generate an aptamer-DOX complex. The complex was able to carry DOX into MUC1 positive tumor cells but lowered the toxicity to MUC1 negative cells. This indicates that the aptamer MA3 has the potential to be used for the targeted delivery of a therapeutic agent to MUC1 expressing tumors^36^. Moreover, there are several aptamers able to internalize cells and inhibit proliferation or induce death. Wang et al.^37^ studied the effects on cell viability of a non-small lung cancer cell cancer (NSCLC)-specific RNA aptamer generated by in vivo SELEX. The chemically synthesized RNA aptamer and truncated form were able to inhibit the cell viability of NCI-H460 cells in a dose-dependent manner, exhibiting IC_50_ values of 118.4 nM and 105.7 nM, respectively^37^. In this sense, our Apt1 could be further explored as a possible cell death inducer due to the significant decrease of cell viability observed.

Besides the proved aptamer's ability to recognize MDA-MB-231 cells, the ability to target breast cancer tissues was also evaluated. A notorious green fluorescence signal was found for Apt2, reinforcing and supporting the potential translation of Apt2 to clinical applications, which could be of great significance in TNBC treatment and detection. The ability of aptamers to recognize human cancer tissues has also been demonstrated by other researchers. Wang and co-workers^38^ described an aptamer against the receptor glucagon identified by cell-SELEX able to bind to the cell membrane of hepatic tissues. Duan and collaborators^39^ identified a DNA aptamer that could be used for imaging clinical metastatic prostate cancer tissues.

## Conclusions

In summary, we have successfully demonstrated a combined analysis pipeline to screen aptamers using cell-SELEX aided by a high throughput aptamer identification method to increase the success of aptamers selection against the target MDA-MB-231 cells. The selected aptamers obtained after 8 rounds of evolved enrichment were further aligned and the 25 most frequent oligonucleotide sequences were analyzed. Two aptamer candidates, Apt1 and Apt2, were characterized regarding their binding affinity to the target, with $K_{d}$ values of 44.3 ± 13.3 and 17.7 ± 2.7, respectively. Both aptamers seem to be associated with cell membrane epitopes only present in the target cells and these aptamers can be subsequently internalized. Apt1 and Apt2 toxicity were assessed showing little to no effect on cells, respectively. The potential translation to clinical applications was proven for Apt2 by immunofluorescence staining of tissue sections, thus highlighting its future potential as a ligand for targeted delivery in MDA-MB-231 TNBC tumors.

## Material and methods

### Cell lines and cell culture

Human breast cancer MDA-MB-231 (ATCC HTB-26) and non-tumorigenic epithelial human breast MCF-10-2A (ATCC CRL-10781) cell lines were used for the cell-SELEX as target cells and negative control, respectively. Hs 578T (ATCC HTB-126), MDA-MB-468 (ATCC HTB-132), MDA-MB-453 (ATCC HTB-131), MCF-7 (ATCC HTB-22) and MDA-MB-157 (ATCC HTB-24) cell lines were also used to confirm the aptamers binding specificity. MDA-MB-231, Hs 578T, MDA-MB-468, MDA-MB-453, MCF-7 and MDA-MB-157 cells were cultured in Dulbecco’s Modified Eagle Medium (DMEM) [Biochrom] supplemented with 10% Fetal Bovine Serum (FBS) [Biochrom] and 1% penicillin–streptomycin [Biochrom] on tissue culture treated flasks. MCF-10-2A cells were cultured in a 1:1 mixture of DMEM:Ham’s F12 medium [Biochrom] supplemented with 20 ng/ml Epidermal Growth Factor (EGF) [Sigma], 100 ng/ml cholera toxin [Sigma], 0.01 mg/ml insulin [Biochrom], 500 ng/ml hydrocortisone [Sigma], 5% horse serum [Biochrom] and 1% penicillin–streptomycin. Cells were propagated at 37 °C, 5% CO_2_ and 95% relative humidity. The cells were washed using phosphate-buffered saline (PBS) 1X pH 7.4 and were detached using Trypsin/EDTA solution [Biochrom]. Cells were counted in a hemocytometer using the trypan blue exclusion test.

### Library, primers and buffers

A randomized ssDNA library with 49 nucleotides (nt) flanked by constant primer binding regions on both sides was used for the cell-SELEX (SB: GCCTGTTGTGAGCCTCCTAAC-nt49-CATGCTTATTCTTGTCTCCC). The polymerase chain reaction (PCR) amplification was performed using a fluorescein labelled forward primer containing 21 nucleotides with an 18-carbon ethylene glycol spacer (P1-F: 5′-FAM-[spacer-C18]-GCCTGTTGTGAGCCTCCTAAC-3′), and a 5′ end phosphate group reverse primer containing 20 nucleotides (P2-Ph: 5′-Phos-GGGAGACAAGAATAAGCATG-3′). The fluorescein label enables the monitoring of the selection progress by flow cytometry and the antisense strand 5′ end with phosphate group helps the ssDNA preparation after each cell-SELEX cycle. Primers and library were HPLC-purified and purchased from Ella Biotech GmbH (Table S1). The binding buffer (BB) was prepared by supplementing PBS 1X with 1 mM of CaCl_2_·2H_2_O [Panreac] and 0.5 mM of MgCl_2_·6H_2_O [VWR]. The washing (WB) and blocking buffers (BlB) were prepared from the BB by adding 0.02 mg/mL or 0.1 mg/mL bovine serum albumin (BSA) [Nzytech], respectively.

### Cell-SELEX procedure

About 2 × 10^5^ cells were plated on 6-well plates two days before the cell-SELEX. At the day of experiment, the culture medium was removed and cells were washed twice with BB and then incubated for 1 min with BlB. The ssDNA library (2 nmol) was prepared in BB, denatured for 5 min at 95 °C, and cooled to down at room temperature for 10 min.

For the counter-selection, the initial library was incubated with MCF-10-2A cells for 30 min at 37 °C with gentle shaking. The library solution was collected and centrifuged at 0.3 g for 3 min and the supernatant was used for the selection step using the target MDA-MB-231 cells for 30 min at 37 °C with gentle shaking. After incubation, the supernatant was removed, and cells were washed with WB to remove unbound sequences. Cells were harvested from the well plate. Cells were heated for 10 min at 95 °C in DNAse-free water and cell-bound ssDNA oligonucleotides were amplified by PCR. Round by round the cell-SELEX stringency was augmented by increasing the number of washes after selection and reducing the number of cells for positive selection (Table S2).

### ssDNA preparation

After each selection round, PCR reactions were performed on microtubes containing 100 µL of PCR solution. Eluted ssDNA oligonucleotides obtained from each cycle were amplified using PCR MasterMix S [PeqLab] and primers (P1-F and P2-Ph) at a final concentration of 0.7 µM under the following conditions: 95 °C for 3 min, 15–25 cycles of 45 s at 95 °C, 45 s annealing at 78 °C and 1 min extension at 72 °C, followed by a final extension of 5 min at 72 °C. The PCR product was purified by MiniElute PCR purification kit [Qiagen]. The final dsDNA in the PCR purified product was separated by Lamba exonuclease digestion [New England Biolabs]^40^. About 6.6 µg of purified dsDNA was incubated at 37 °C for 2 h with 25U of lambda exonuclease in 50 µL of total reaction volume. The reaction was stopped by incubation at 75 °C for 10 min. Purified PCR products were analyzed on a 10% denaturing urea-polyacrylamide gel and stained with GelRed [Biotium].

### Sequencing and data analysis

The resulting ssDNA pool (from the 8th cycle) with the highest degree of enrichment was sequenced using Illumina MiSeq by Fasteris (Switzerland). The pool for sequencing was prepared by performing an overlap PCR with primers that are complementary to constant regions of the randomized oligonucleotide library with an added overhang that includes an Illumina platform-specific sequence. Afterwards, the sequencing reads were filtered and demultiplexed. For this analysis, constant primer binding regions were removed and sequences longer or shorter than 49 nucleotides were discarded. Raw next-generation sequencing (NGS) data was analyzed using Matlab R2019a (The Mathworks, Inc.) and the alignment algorithm embedded in Geneious 9.1.4 software (Biomatters Ltd.; https://www.geneious.com/). The 25 most frequent oligonucleotide sequences were further evaluated. The phylogenetic tree was constructed using Tree Builder function in Geneious, where the tree distances and sequence relatedness was determined by the method of Tamura and Nei using a neighbor-joining model with no outgroup. The free energy (∆G) and secondary structure were calculated using the Mfold software, presenting the optimal structure for each aptamer^23^. The conditions used for the structure predictions were set according to the constituents of the BB at 37 °C in 187 mM Na^+^ and 0.5 mM Mg^2+^ at pH 7.4.

### Binding experiments

#### Flow cytometry

To monitor the cell-SELEX progress and the binding ability of the selected aptamers, flow cytometry analysis was performed. About 250 nM of FAM-labelled ssDNA pools or aptamers were incubated with 0.5 × 10^5^ target or negative (control) cells at 37 °C for 30 min. Cells were then washed, harvested, and finally resuspended in BB. The FAM-labelled ssDNA library (SB-F) was used as negative control.

To determine the equilibrium dissociation constant ($K_{d}$), MDA-MB-231 and MCF-10-2A cells were incubated with concentrations of FAM-labelled aptamers (Apt1-F and Apt2-F) ranging from 0 to 400 nM, in 200 µL of BB at 37 °C for 30 min. SB-F was used as negative control. Cells were washed and harvested by using a cell scraper and then resuspended in 200 µL of BB for flow cytometry analysis. The $K_{d}$ of the aptamer-cell interaction was determined by plotting the average total percentage of fluorescent cells (Y), against the concentration of aptamer (X, nM) and the B_max_ as the maximum number of binding sites, using a non-interacting binding sites model according to Eq. (1):

1 $$Y = \frac{{B_{max} X}}{{\left( {K_{d} + X} \right)}},$$

through GraphPad Prism (GraphPad Prism version 7.00 for Windows).

To evaluate the specificity of the selected aptamers, binding assays with other breast cancer cell lines (Hs 578T, MDA-MB-157, MDA-MB-468, MDA-MB-453 and MCF-7) were performed. The fluorescence signal was measured using an EC800 flow cytometer analyzer [Sony Biotechnology] by counting at least 20,000 events.

#### Fluorescence microscopy

Fluorescence microscopy imaging was performed to monitor the binding affinity of the selected aptamers to live cells. About 2 × 10^5^ cells were seeded over coverslips in 6-well plates and cultured overnight. Subsequently, the cells were washed twice and incubated with 250 nM of Apt1-F and Apt2-F in BB at 37 °C for 30 min. After washing with 500 µL WB, the cells were fixed with 4% paraformaldehyde (PFA) [Sigma] for 40 min at room temperature. After fixation, cells were once again washed of 4′,6-diamidino-2-phenylindole (DAPI) [Biotium] was added and incubated for 15 min at room temperature. The coverslips were placed in slides and the cells were observed in a fluorescence microscope [OLYMPUS BX51] incorporated with a high-sensitivity camera Olympus DP71 at 10 × or 20 × magnification.

### Effects of temperature and proteinase/trypsin treatment on aptamer binding ability

To perform a target-type analysis, MDA-MB-231 cells were treated with 0.1 mg/mL Proteinase K [Fisher BioReagents] or 0.25% trypsin [Sigma] solution for 3 or 10 min. In both cases, the incubation occurred at 37 °C and the reaction was stopped by adding 100 µL of DMEM with 10% (v/v) of FBS. Then, cells were washed twice and incubated with 250 nM of Apt1-F and Apt2-F at 37 °C for 30 min. After washing with WB, cell samples were analyzed by flow cytometry.

To ascertain the effect of temperature on the aptamer binding ability, MDA-MB-231 cells were incubated with 250 nM of FAM-labelled Apt1 and Apt2 at 4 °C or 37 °C for 30 min. After washing with WB, cell samples were analyzed by flow cytometry under the conditions previously described.

### In vitro cytotoxicity assay

Cell viability was evaluated using the 3-(4, 5-dimethylthiazol-2-yl)-2, 5-diphenyltetrazolium bromide) (MTT) assay [Sigma]. MDA-MB-231 and MCF-10-2A cells (1 × 10^4^ cells/well) were plated onto a 96-well plate in the day before the experiment. Cells were incubated for 24 and 48 h at 37 °C with 250 nM of both aptamers, which were previously resuspended in BB, boiled at 95 °C for 5 min, and then cooled for 10 min at room temperature. SB was used as a negative control. After the incubation time, 100 µL MTT solution (0.5 mg/mL) was added to each well and cells were incubated at 37 °C for 4 h. Then, the MTT solution was removed and 150 µL of dimethyl sulfoxide (DMSO) [Sigma] was added to solubilize blue formazan crystals. The absorbance was read at 570 nm in a microplate reader [Cytation 3, BioTek].

### Co-localization studies

About 3 × 10^4^ cells were seeded on coverslips in 24-well plates and cultured overnight. Cells were incubated with Apt1-F and Apt2-F in BB at 37 °C for 30 min and then were washed twice with WB.

For plasma membrane labelling, about 0.01 mg/ml of filipin [Sigma] dissolved in PBS 1X supplemented with 0.5% BSA was added to cells right before imaging.

For actin filaments staining and cell cytoskeleton delineation, cells were fixed with 4% PFA for 40 min at room temperature. After rinsing with PBS 1X, cells were incubated with 50 mM ammonium chloride [Sigma] for 10 min, rinsed again and permeabilized with 0.1% of sodium dodecyl sulfate (SDS) Sigma) in PBS 1X for 10 min. Next, cells were blocked in PBS 1X supplemented with 3% of BSA for 20 min and washed. Following this procedure, cells were incubated with CF 568 Phalloidin [Biotium] for 1 h in the dark in a humidified atmosphere. After incubation, cells were once again washed with PBS 1X with 0.1% of BSA, DAPI added and incubated for 15 min at room temperature. Coverslips were mounted upside down in Vectashield mounting medium and the images were acquired in a fluorescence microscope [OLYMPUS BX51] incorporated with a high-sensitivity camera Olympus DP71, using a at 60X oil immersion objective.

To trace the cellular uptake of the aptamers, 1 mM of lysoSensor Red DND-99 [ThermoFisher] (1.5 µM final concentration) was added to each sample and incubated during 30 min at 37 °C. Next, the medium was removed and the coverslips were mounted upside down. Images were acquired in a sequential mode by a confocal scanning laser microscope (BX61 FLUOVIEW1000, Olympus), using a 60X oil immersion objective and with the specific filter settings for FAM (laser excitation line 488 nm and emissions filters BA 505–540, green channel) and RED DND-99 dye (laser excitation line 559 nm and emissions filters BA 575–675, red channel).

### Fluorescence staining of breast cancer tissue sections

Tumor tissue fluorescence staining was used to confirm the aptamer's ability to bind MDA-MB-231 tissues. Formalin-fixed paraffin-embedded breast cancer tissue sections were deparaffinized, rehydrated and then antigenic retrieval was performed as described elsewhere^41^. Briefly, the slides were heated in 10 mM sodium citrate buffer pH 6.0 [Sigma] at 95 °C for 20 min followed by slow cooling at room temperature for about 20 min. Then, tissue slides were blocked with BlB for 30 min at room temperature. Afterwards, the slides were incubated with 250 nM of Apt1-F, Apt2-F or SB-F in BB for 30 min at 37 °C and washed with WB. Finally, the slides were stained with Vectashield mounting media containing DAPI solution. Images were acquired using an Olympus BX51 microscope incorporated with a high-sensitivity camera Olympus DP71 at 40X magnification.

### Statistical analysis

Data were expressed as mean ± standard deviation (SD) of three independent experiments. One-way ANOVA with Tukey’s post-test and two-way ANOVA with Sidak’s post-test were performed using GraphPad Prism to identify differences among multiple groups, considering a significance level of 95%.

## Supplementary Information

Supplementary Information 1.

Supplementary Information 2.

Supplementary Information 3.

Supplementary Information 4.

## Abbreviations

DAPI: 4′,6-Diamidino-2-phenylindole
DMEM: Dulbecco’s Modified Eagle Medium
DMSO: Dimethyl sulfoxide
DOX: Doxorubicin
EGF: Epidermal growth factor
ER: Estrogen
FBS: Fetal bovine serum
HER2: Epidermal growth factor receptor 2
HTS: High throughput sequencing
$$K_{d}$$: Dissociation constant
MTT: 3-(4, 5-Dimethylthiazol-2-yl)-2, 5-diphenyltetrazolium bromide)
MUC1: Mucin 1
NGS: Next-generation sequencing
NSCLC: Non-small lung cancer cell cancer
PARP: Poly (ADP-ribose) polymerase
PBS: Phosphate-buffered saline
PR: Progesterone
RNA: Ribonucleic acid
SD: Standard deviation
SELEX: Systematic evolution of ligands by exponential enrichment
SL: Stem-loop
ssDNA: Single-stranded deoxyribonucleic acid
TNBC: Triple-negative breast cancer
