# Supplementary figures and images for: Selection of aptamers against triple negative breast cancer cells using high throughput sequencing
Source: Sci Rep. 2021 Apr 21;11:8614. doi: 10.1038/s41598-021-87998-y (PMC8060331; doi:10.1038/s41598-021-87998-y)

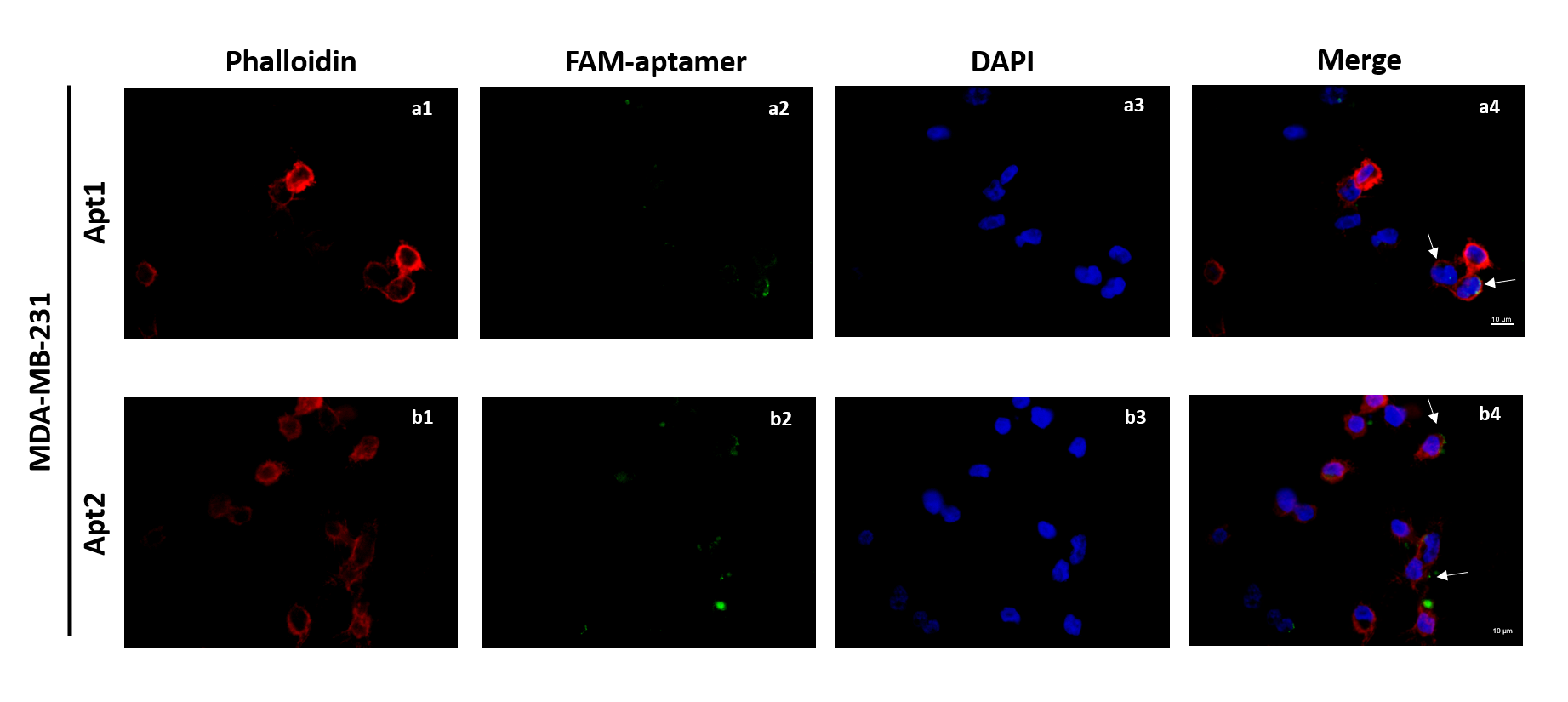

Supplement: Supplementary file 1 — Supplementary Information 1. [file 41598_2021_87998_MOESM1_ESM.tiff]

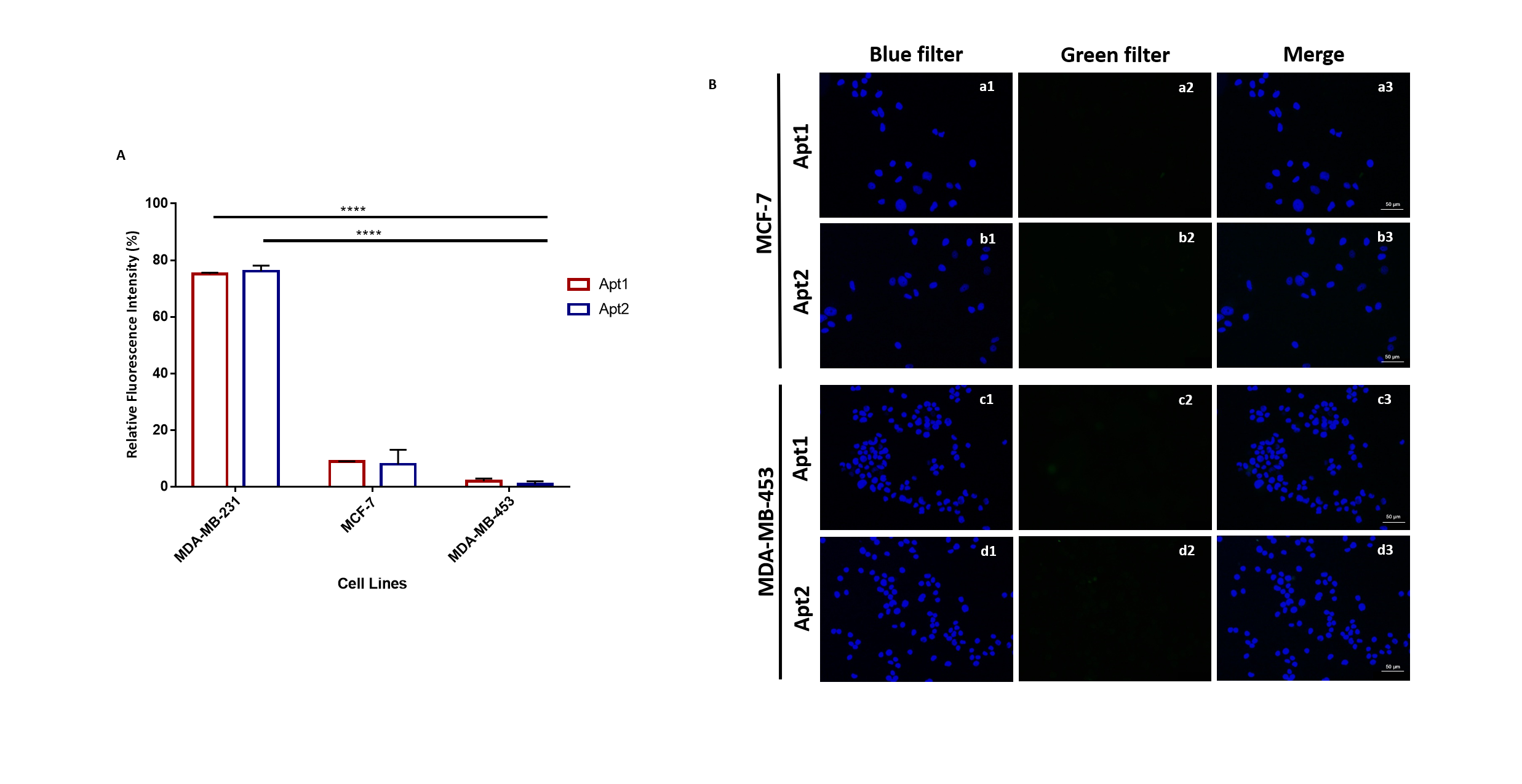

Supplement: Supplementary file 2 — Supplementary Information 2. [file 41598_2021_87998_MOESM2_ESM.tiff]
