# Supplementary material for: Selection of aptamers against triple negative breast cancer cells using high throughput sequencing
Source: Sci Rep. 2021 Apr 21;11:8614. doi: 10.1038/s41598-021-87998-y (PMC8060331; doi:10.1038/s41598-021-87998-y)
Supplement: Supplementary file 3 — Supplementary Information 3. [file 41598_2021_87998_MOESM3_ESM.docx]

**Selection of aptamers against triple negative breast cancer cells using high throughput sequencing**

Débora Ferreira, ^1,2,†^ Joaquim Barbosa,^1,2,†^ Diana A. Sousa,^1,2^ Cátia Silva, ^1^ Luís D.R. Melo ^1^, Meltem Avci-Adali,^3^ Hans P. Wendel ^3^ and Ligia R. Rodrigues ^1,2,^*

* Corresponding author: Tel: (+351) 253601978, Email: [lrmr@deb.uminho.pt](mailto:lrmr@deb.uminho.pt)

**Running Title:** Aptamers targeting triple-negative breast cancer

**Table S1. Oligonucleotide sequences used in the selection and validation process.**

| **ID** | **Name** | **Sequence (5'-3')** |
| --- | --- | --- |
| SB | Start Library | GCCTGTTGTGAGCCTCCTAAC-**nt49**-CATGCTTATTCTTGTCTCCC |
| SB-F | FAM-Library | Fam-C18 Spacer-GCCTGTTGTGAGCCTCCTAAC-**nt49**-CATGCTTATTCTTGTCTCCC |
| P1 | Forward Primer | GCCTGTTGTGAGCCTCCTAAC |
| P1-F | FAM-Forward Primer | Fam-C18 Spacer-GCCTGTTGTGAGCCTCCTAAC |
| P2 | Reverse Primer | GGGAGACAAGAATAAGCATG |
| P2-Ph | Phosphorylated-Reverse Primer | Phosphate-GGGAGACAAGAATAAGCATG |
| P2-Bio | Biotin-Reverse Primer | Biotin-GGGAGACAAGAATAAGCATG |
| Apt1 | Aptamer 1 | GCCTGTTGTGAGCCTCCTAAC**GCATCCACCGTGAATATTGTAACGCTATATGTGAGTGGCTAAGTGCACC**CATGCTTATTCTTGTCTCCC |
| Apt1-F | FAM-Aptamer 1 | Fam-C18 Spacer-GCCTGTTGTGAGCCTCCTAAC**GCATCCACCGTGAATATTGTAACGCTATATGTGAGTGGCTAAGTGCACC**CATGCTTATTCTTGTCTCCC |
| Apt2 | Aptamer 2 | GCCTGTTGTGAGCCTCCTAAC**ATGTTGTTGCCGGGACGCCTCCTTCACCAAAGTTGGTGTCCCCACCTAC**CATGCTTATTCTTGTCTCCC |
| Apt2-F | FAM-Aptamer 2 | Fam-C18 Spacer-GCCTGTTGTGAGCCTCCTAAC**ATGTTGTTGCCGGGACGCCTCCTTCACCAAAGTTGGTGTCCCCACCTAC**CATGCTTATTCTTGTCTCCC |

**Table S2. Conditions used in the different selection rounds**. The selection process was stopped after 18^th^ selection rounds.

| **Rounds** | **Selection**  **(cell number)** | **Counter-Selection**  **(cell number)** | **Washes**  **(after selection)** | **PCR**  **(cycles)** |
| --- | --- | --- | --- | --- |
| 1 | 2.E+05 | -- | 1 | 25 |
| 2 | 2.E+05 | 2.E+05 | 1 | 20 |
| 3 | 2.E+05 | 2.E+05 | 1 | 20 |
| 4 | 2.E+05 | 2.E+05 | 1 | 18 |
| 5 | 2.E+05 | 2.E+05 | 2 | 15 |
| 6 | 2.E+05 | 2.E+05 | 2 | 15 |
| 7 | 2.E+05 | 2.E+05 | 2 | 15 |
| **8** | **1.E+05** | **2.E+05** | **2** | **15** |
| 9 | 1.E+05 | 2.E+05 | 4 | 12 |
| 10 | 1.E+05 | 2.E+05 | 4 | 12 |
| 11 | 1.E+05 | 2.E+05 | 4 | 12 |
| 12 | 1.E+05 | 2.E+05 | 4 | 12 |
| 13 | 5.E+04 | 2.E+05 | 4 | 14 |
| 14 | 5.E+04 | 2.E+05 | 4 | 14 |
| 15 | 5.E+04 | 2.E+05 | 4 | 14 |
| 16 | 5.E+04 | 2.E+05 | 4 | 14 |
| 17 | 5.E+04 | 2.E+05 | 4 | 14 |
| 18 | 5.E+04 | 2.E+05 | 4 | 14 |

### Table S3. Secondary structures of top-ranked selected aptamers. Predicted secondary structures for the other twenty-three aptamer candidates. The presented predicted secondary structures were the ones with lowest ΔG, i.e. the highest stability using the temperature 37 °C, 187 mM Na^+^ and 0.5 mM Mg2^+^ (calculated using the mfold webserver). Constant sequence regions are highlighted in black, and blue represents the random regions.

| **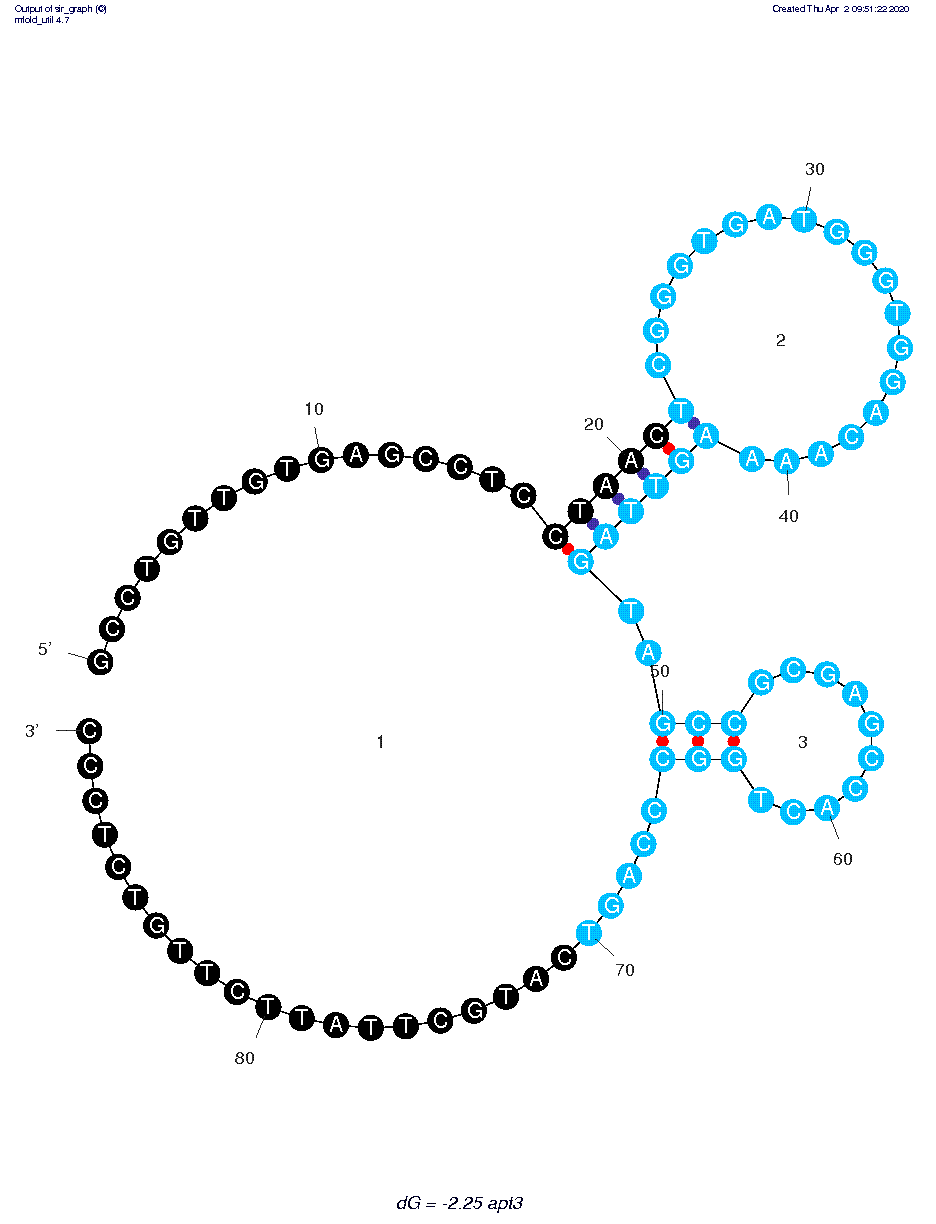**  **Apt3, ∆G = -2.3** | **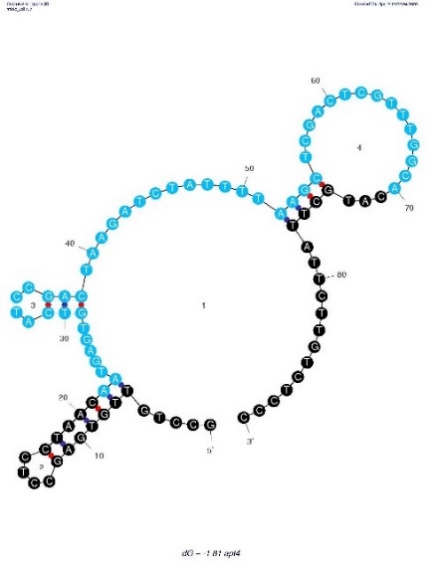**  **Apt4, ∆G = -1.8** | 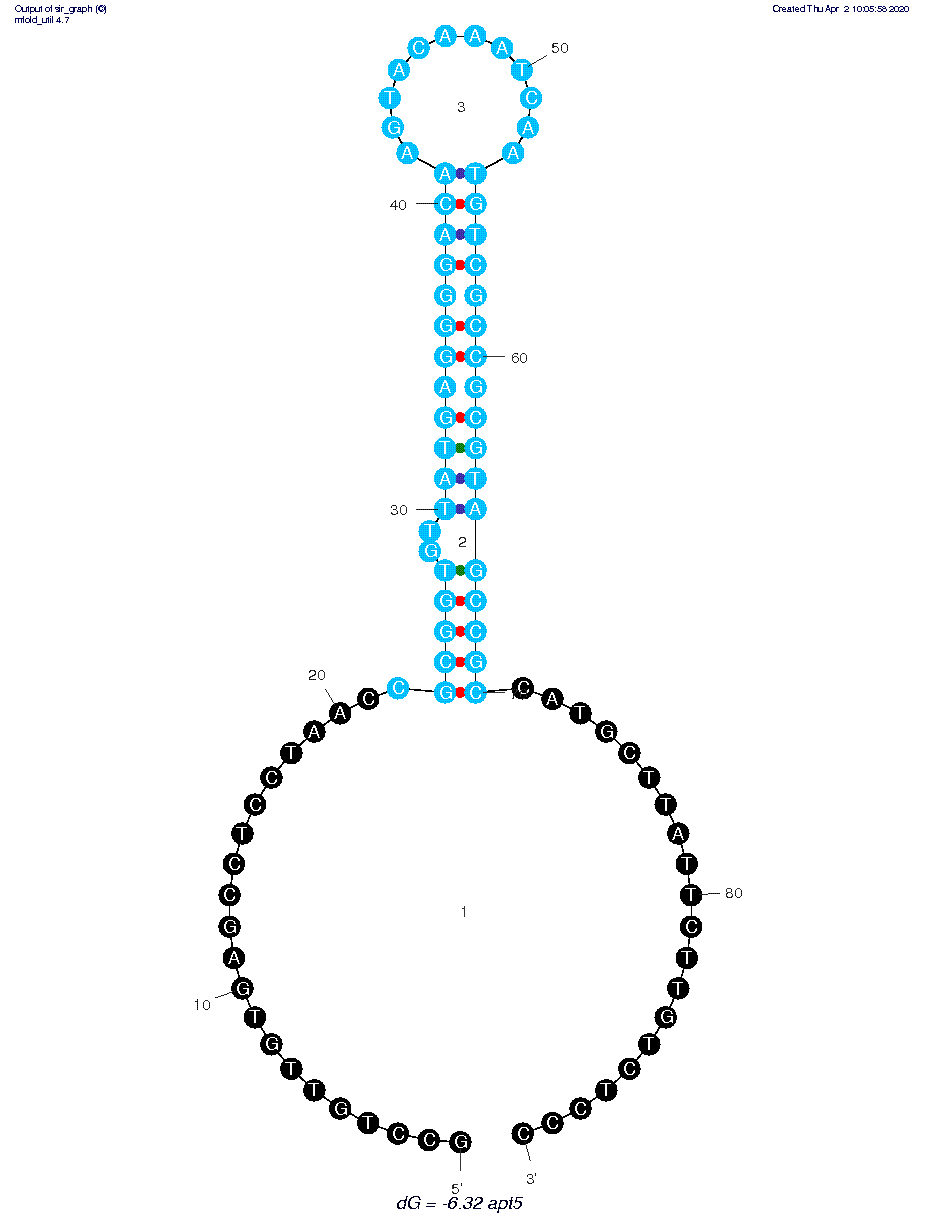  **Apt5, ∆G = -6.3** |
| --- | --- | --- |
| 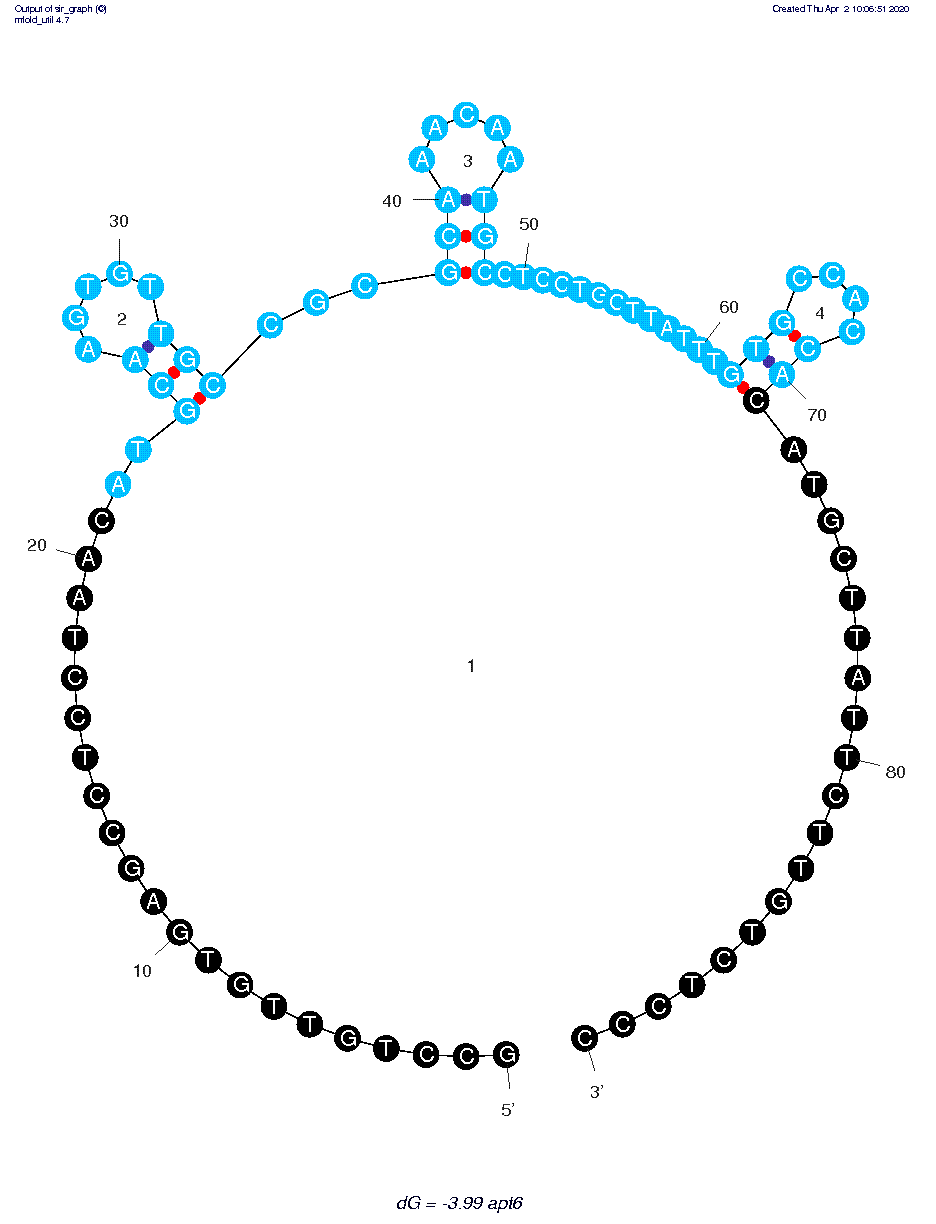  **Apt6, ∆G = -4** | 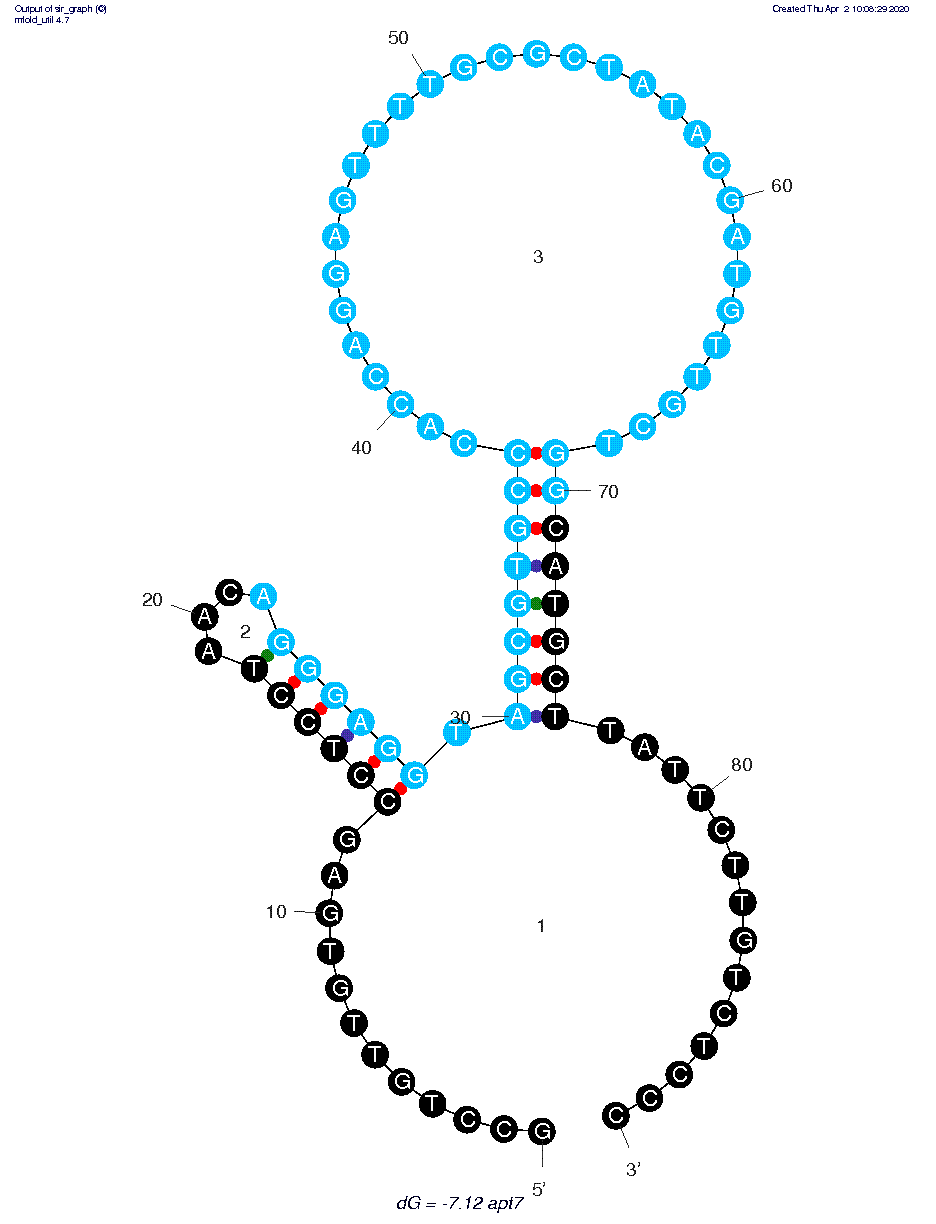  **Apt7, ∆G = -7.1** | 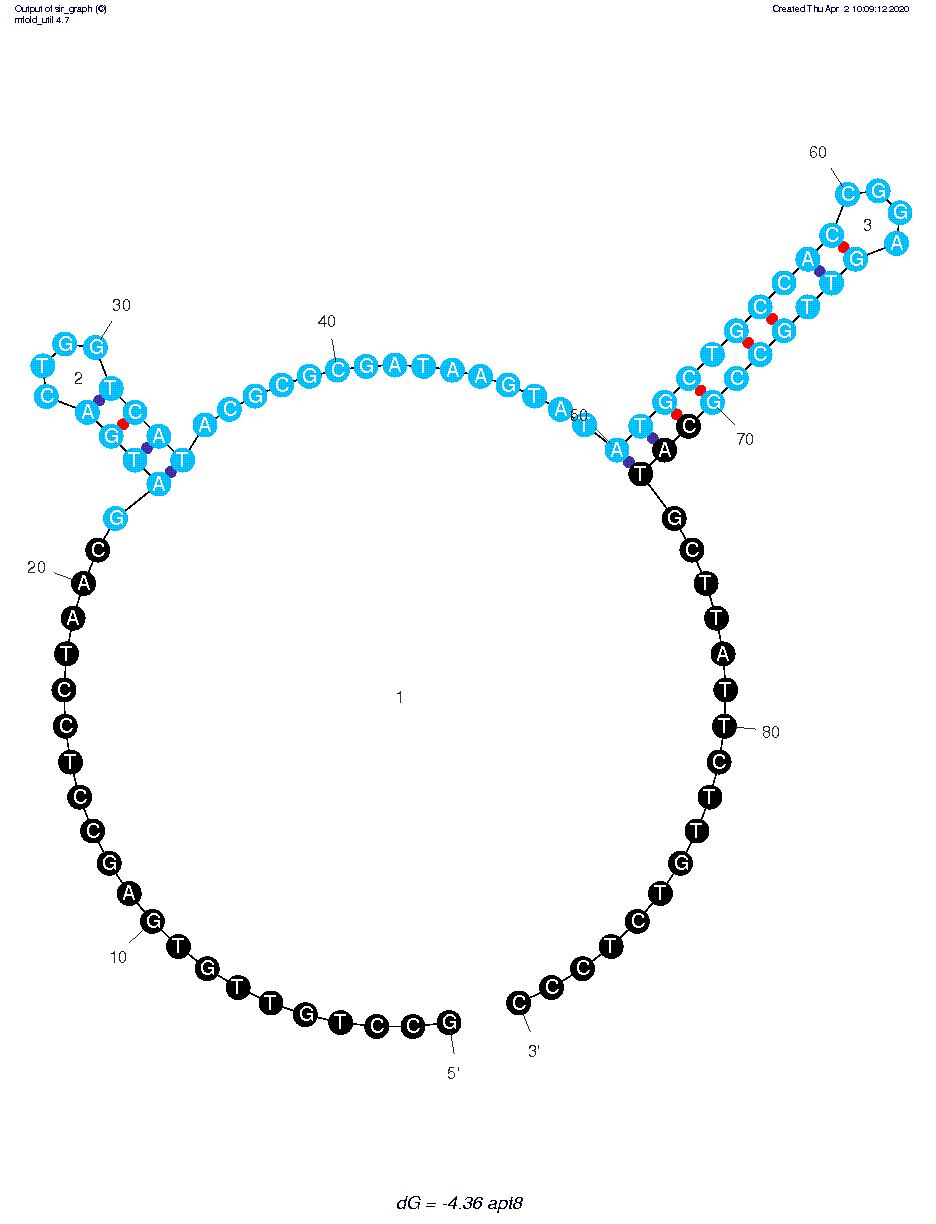  **Apt8, ∆G = -4.4** |
| 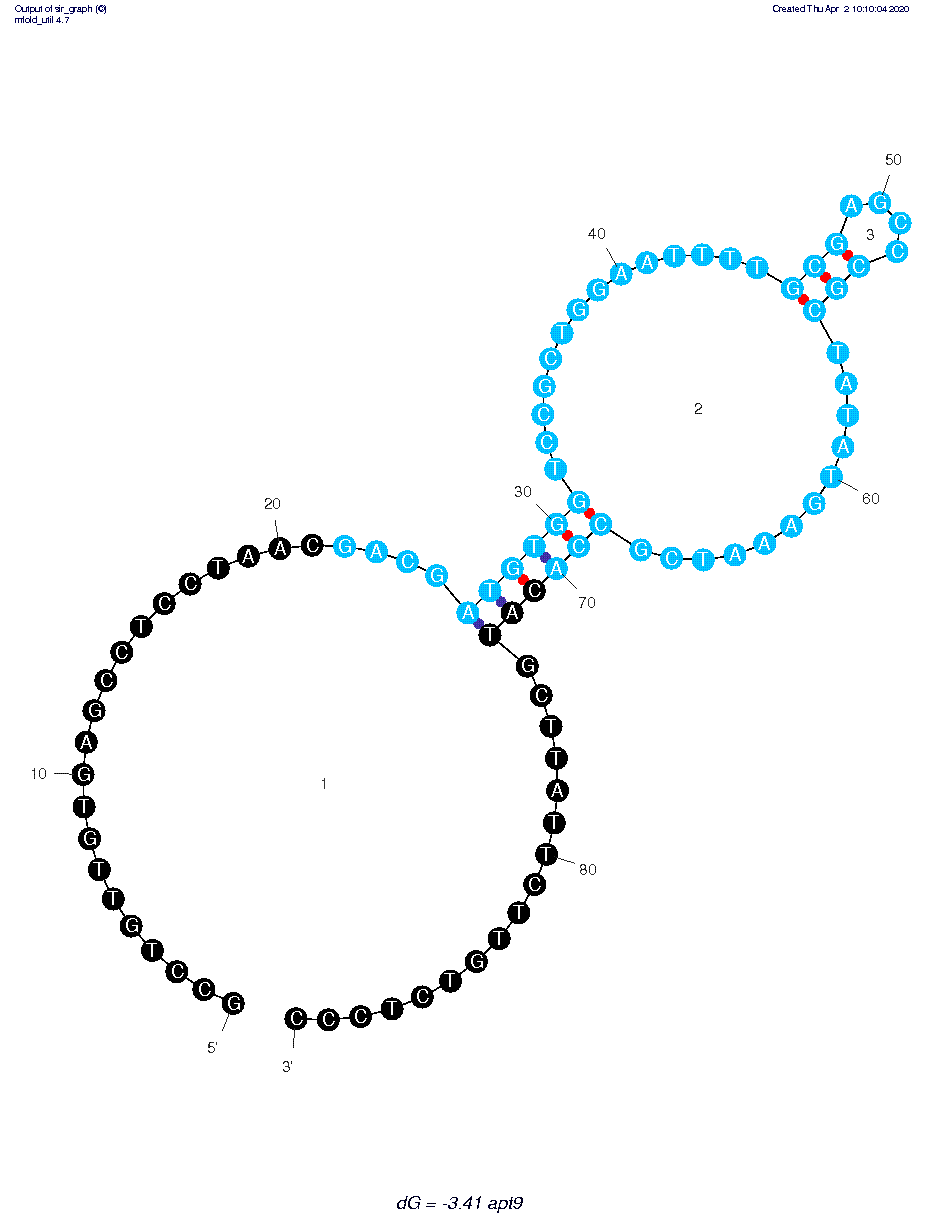  **Apt9, ∆G = -3.4** | 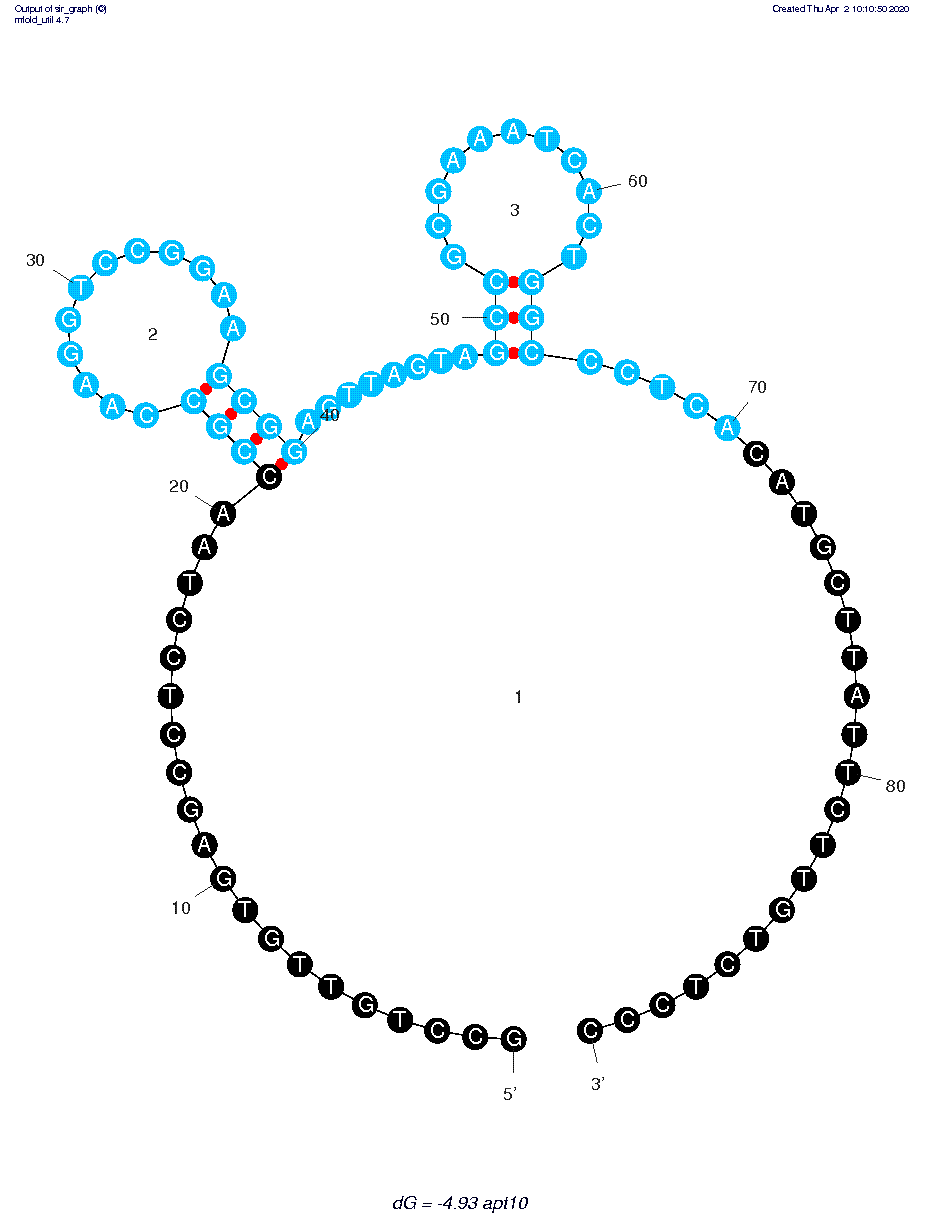  **Apt10, ∆G = -4.9** | 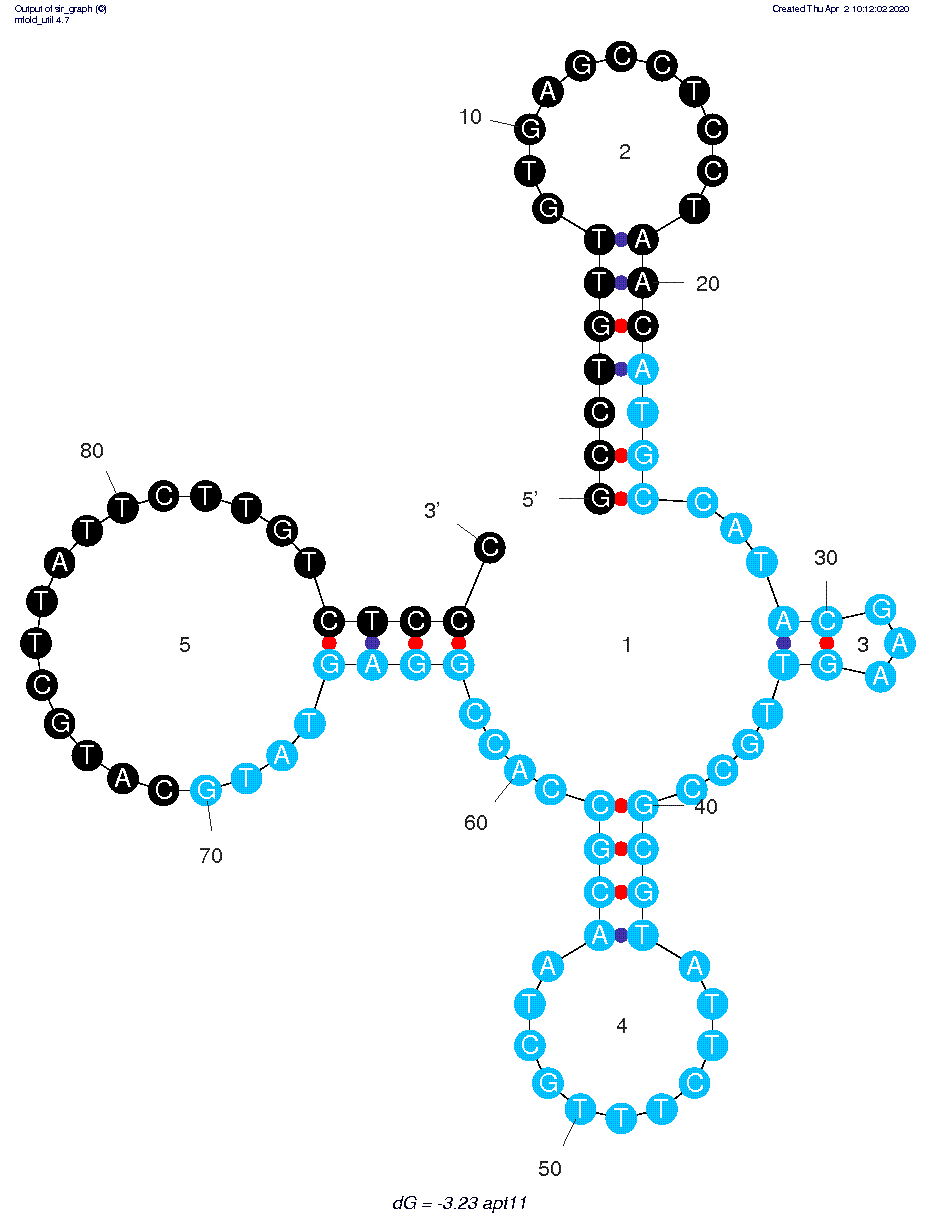  **Apt11, ∆G = -3.2** |
| 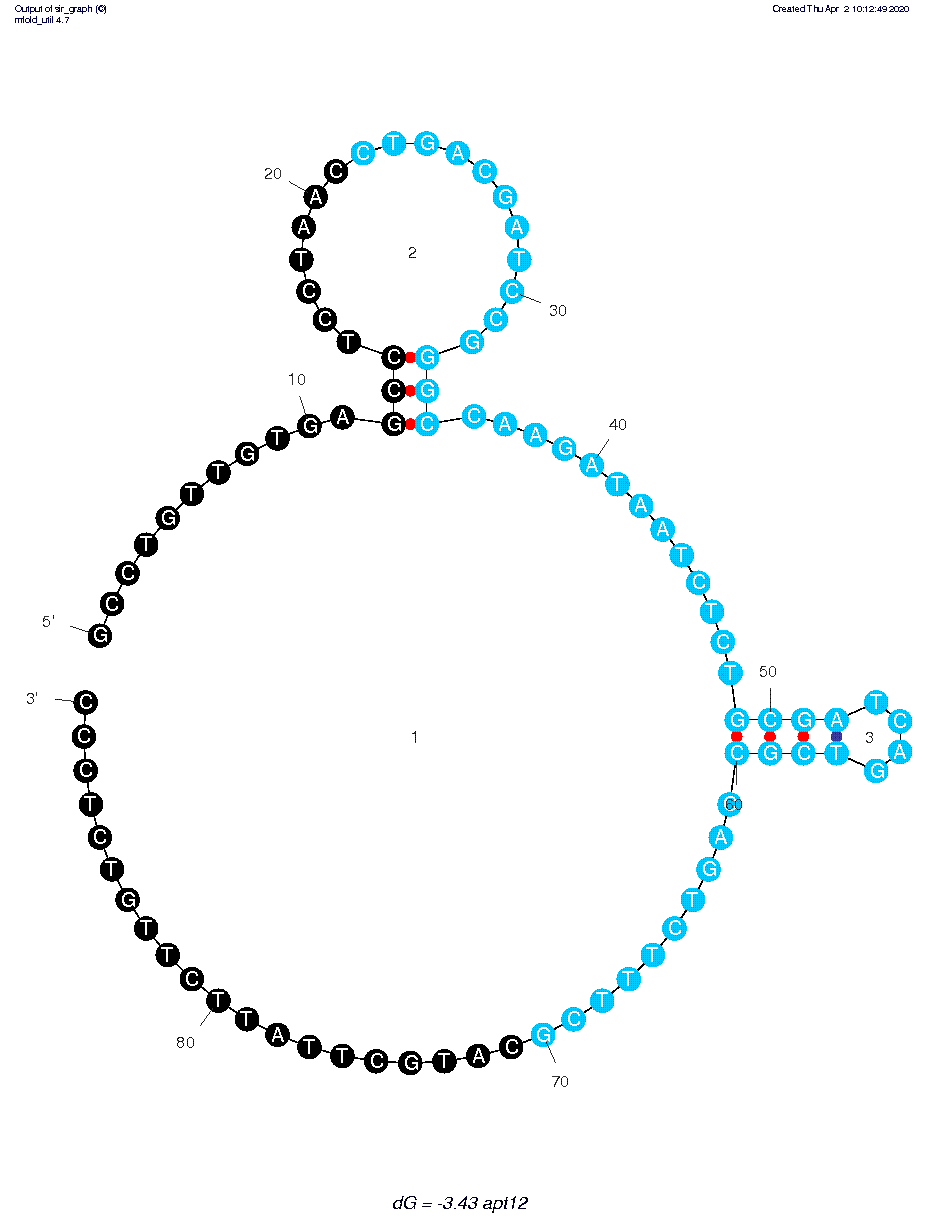  **Apt12, ∆G = -3.4** | 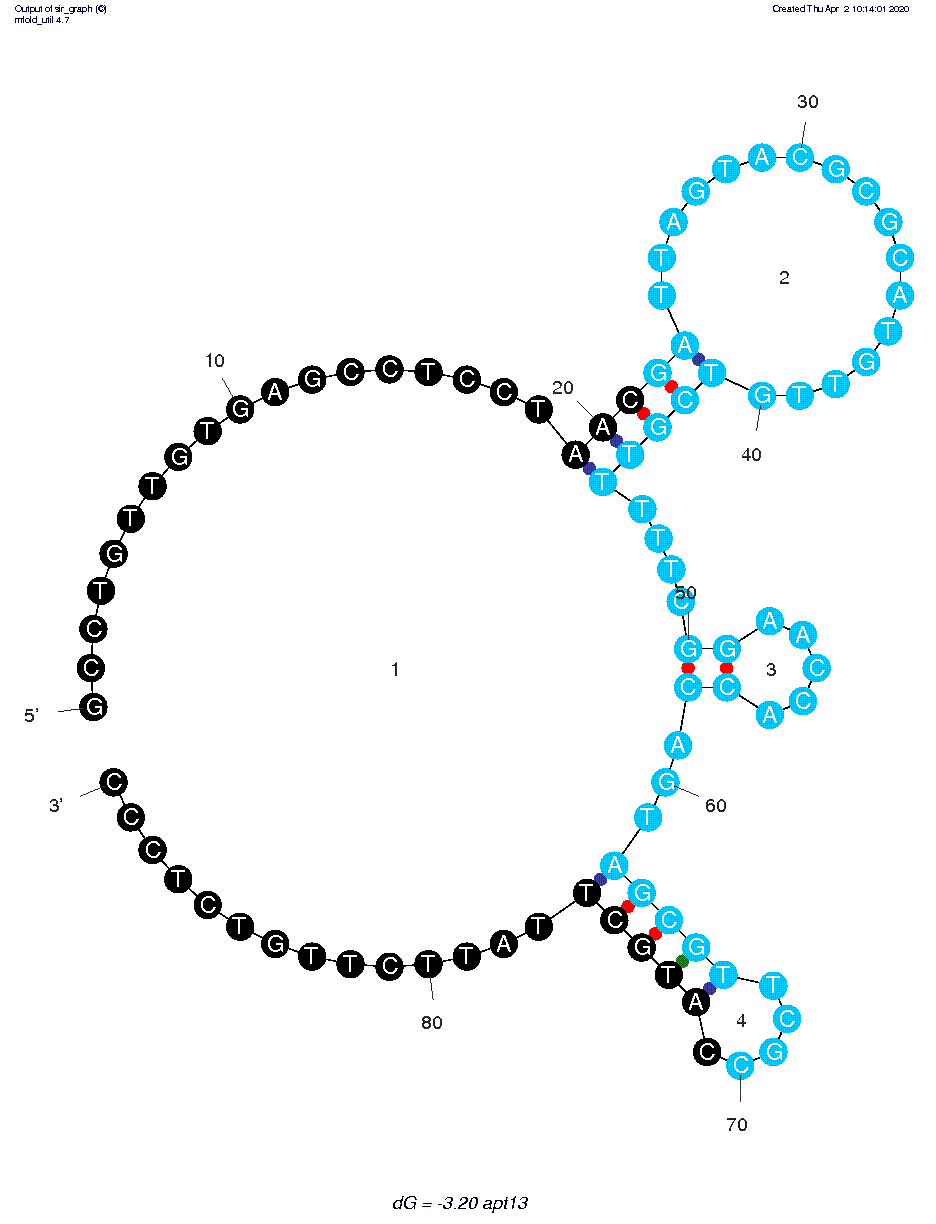  **Apt13, ∆G = -3.2** | 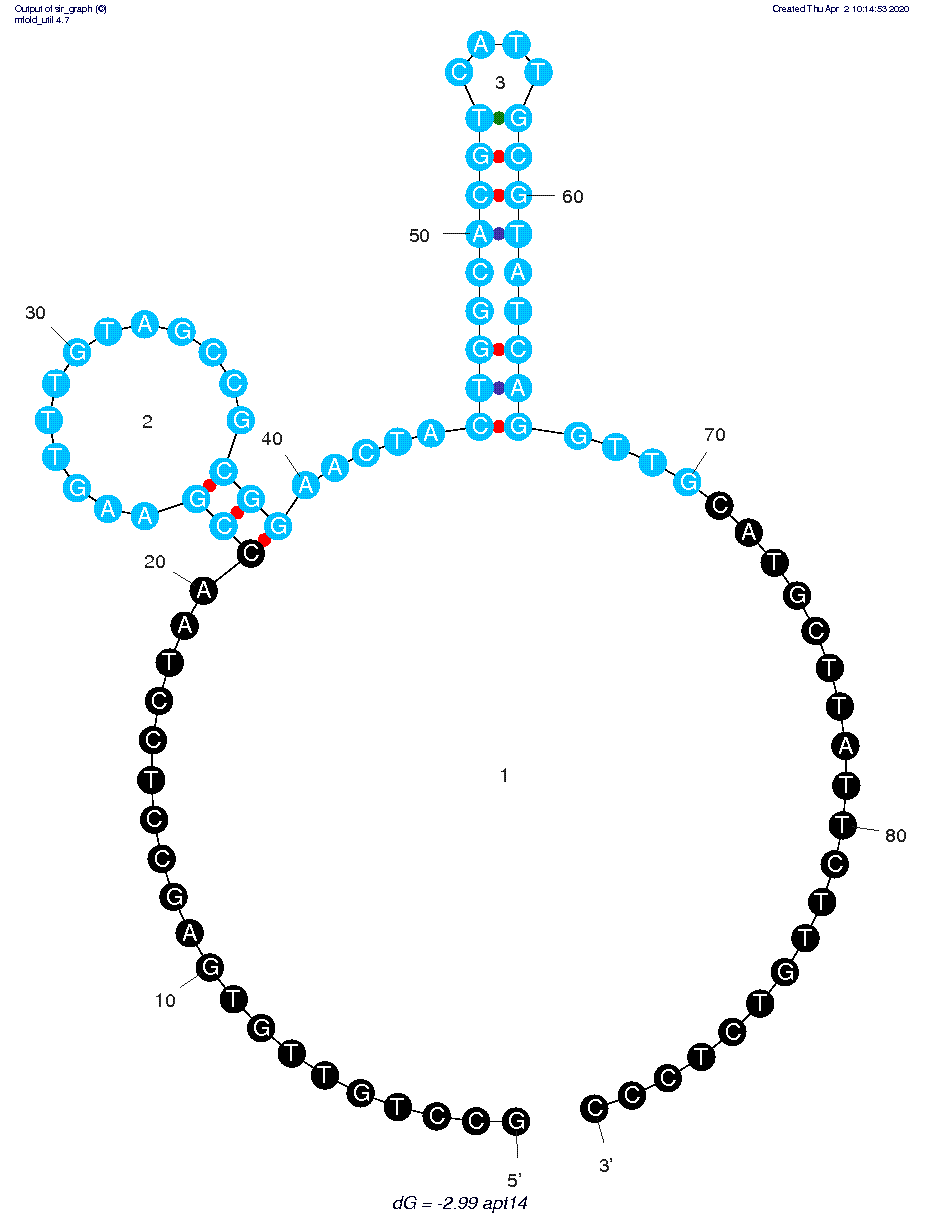  **Apt14, ∆G = -3** |
| 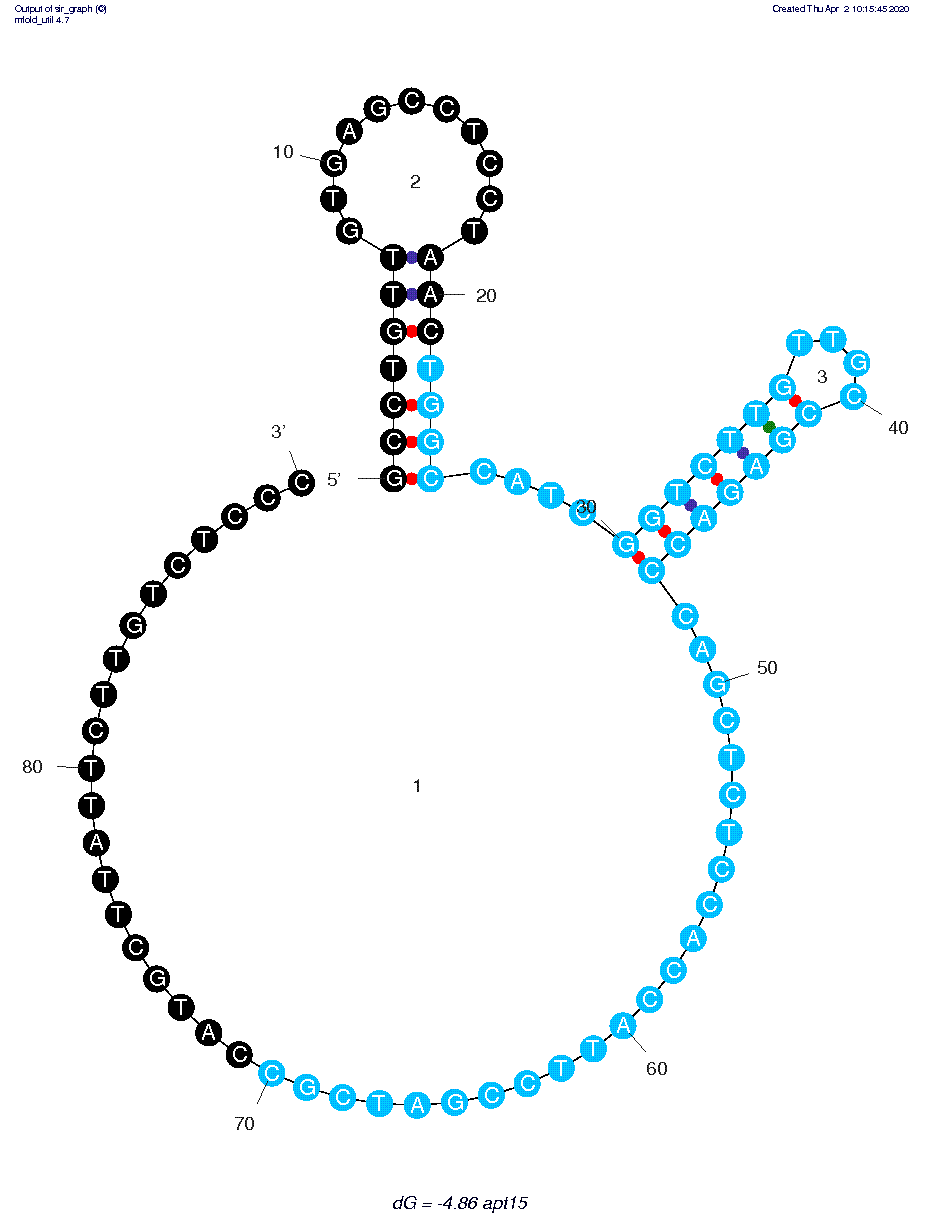  **Apt15, ∆G = -4.9** | 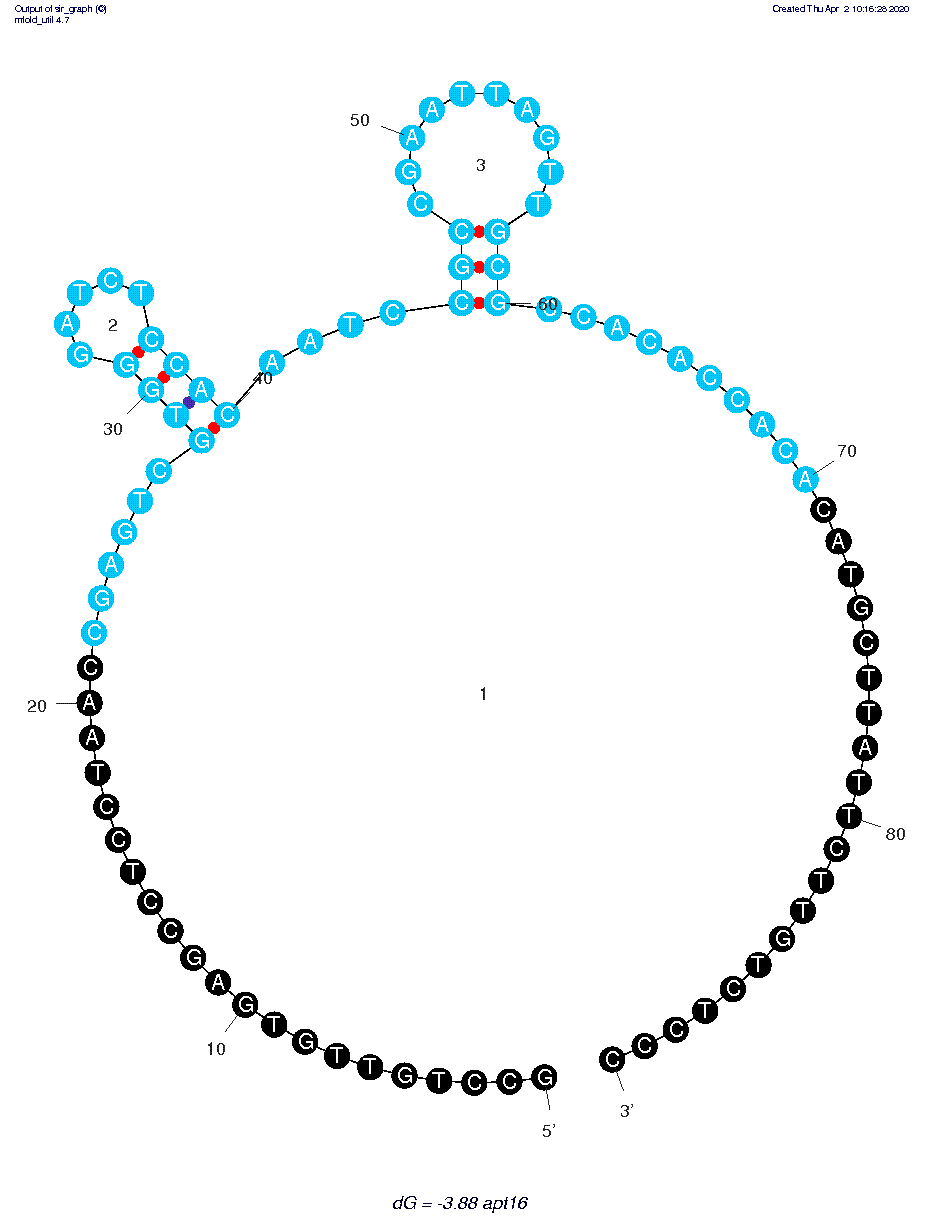  **Apt16, ∆G = -3.9** | 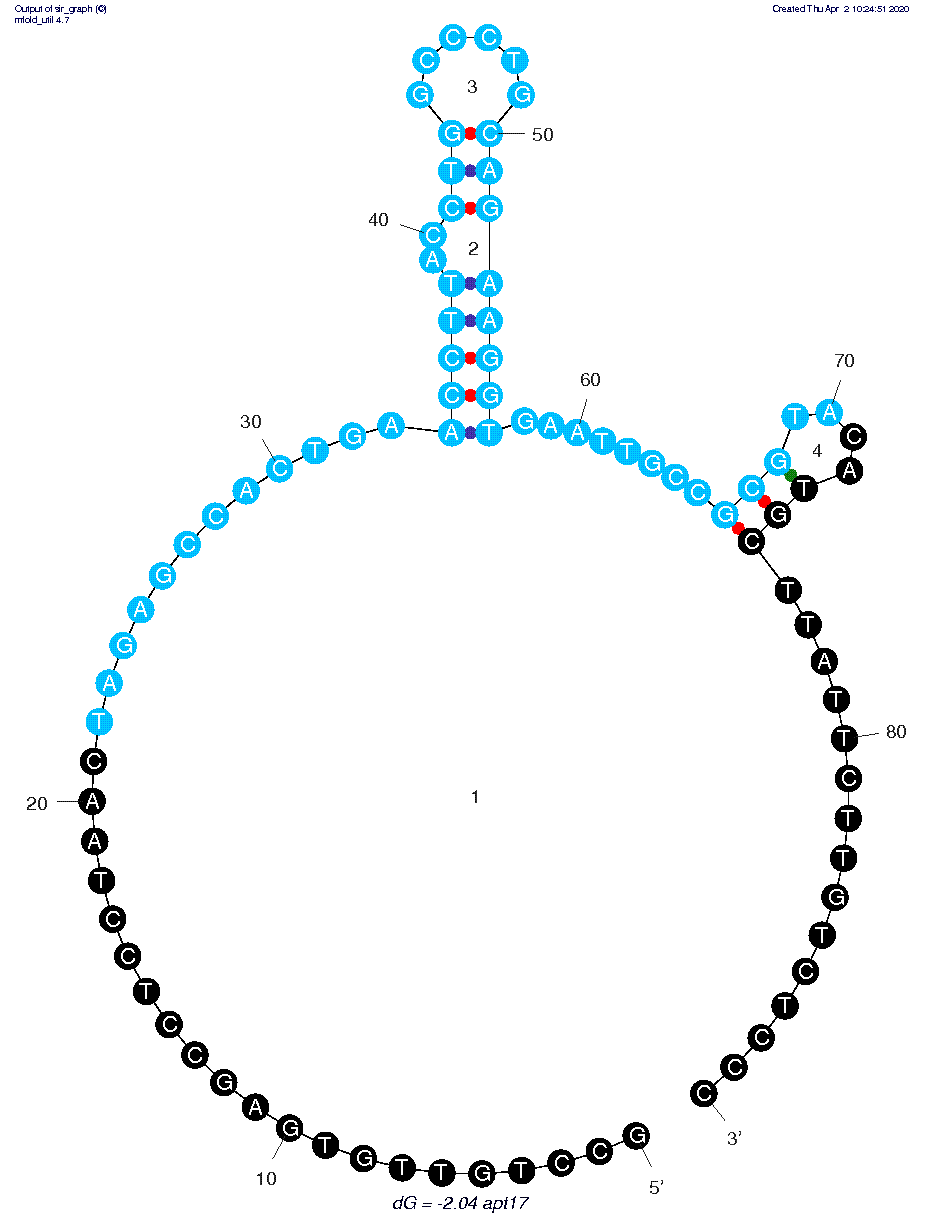  **Apt17, ∆G = -2** |
| 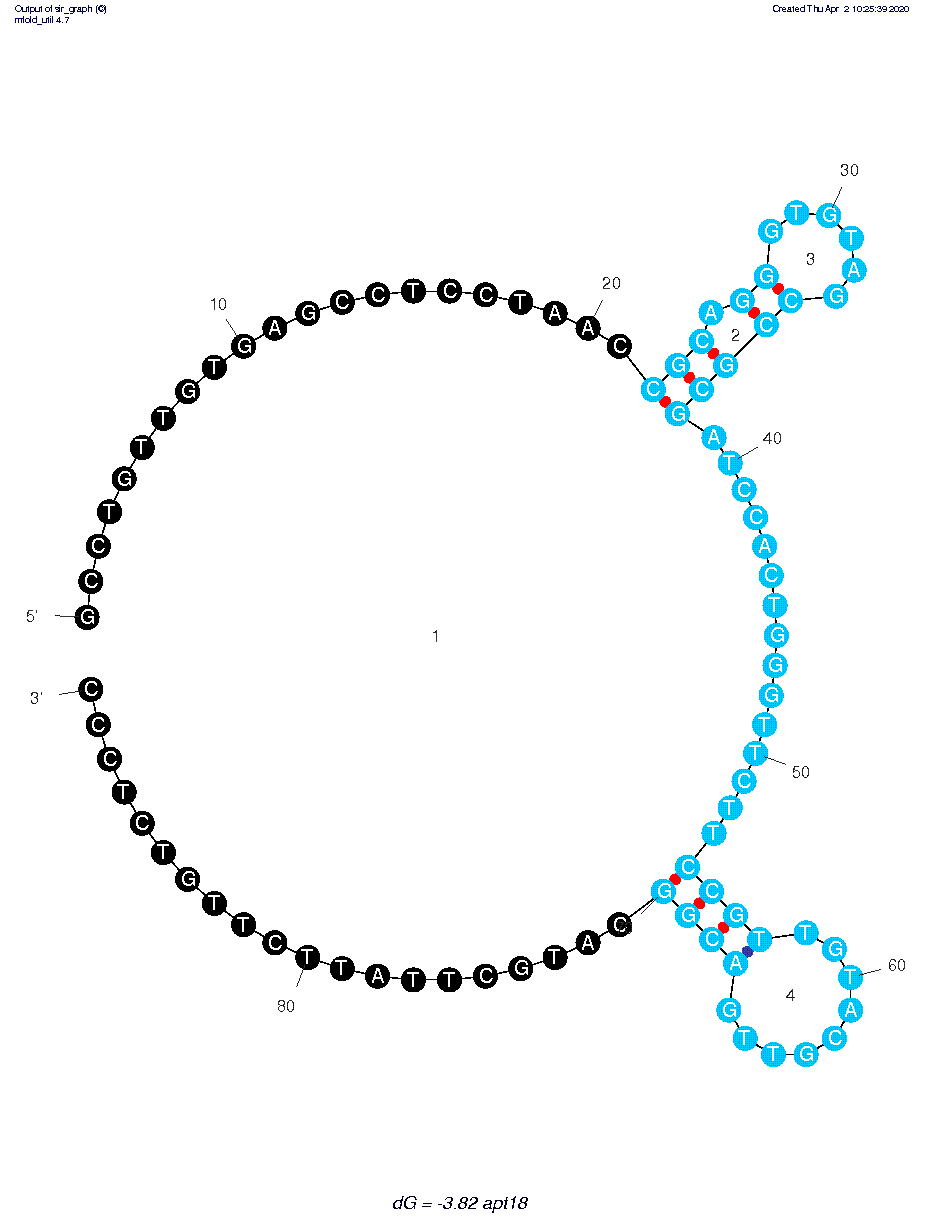  **Apt18, ∆G = -3.8** | 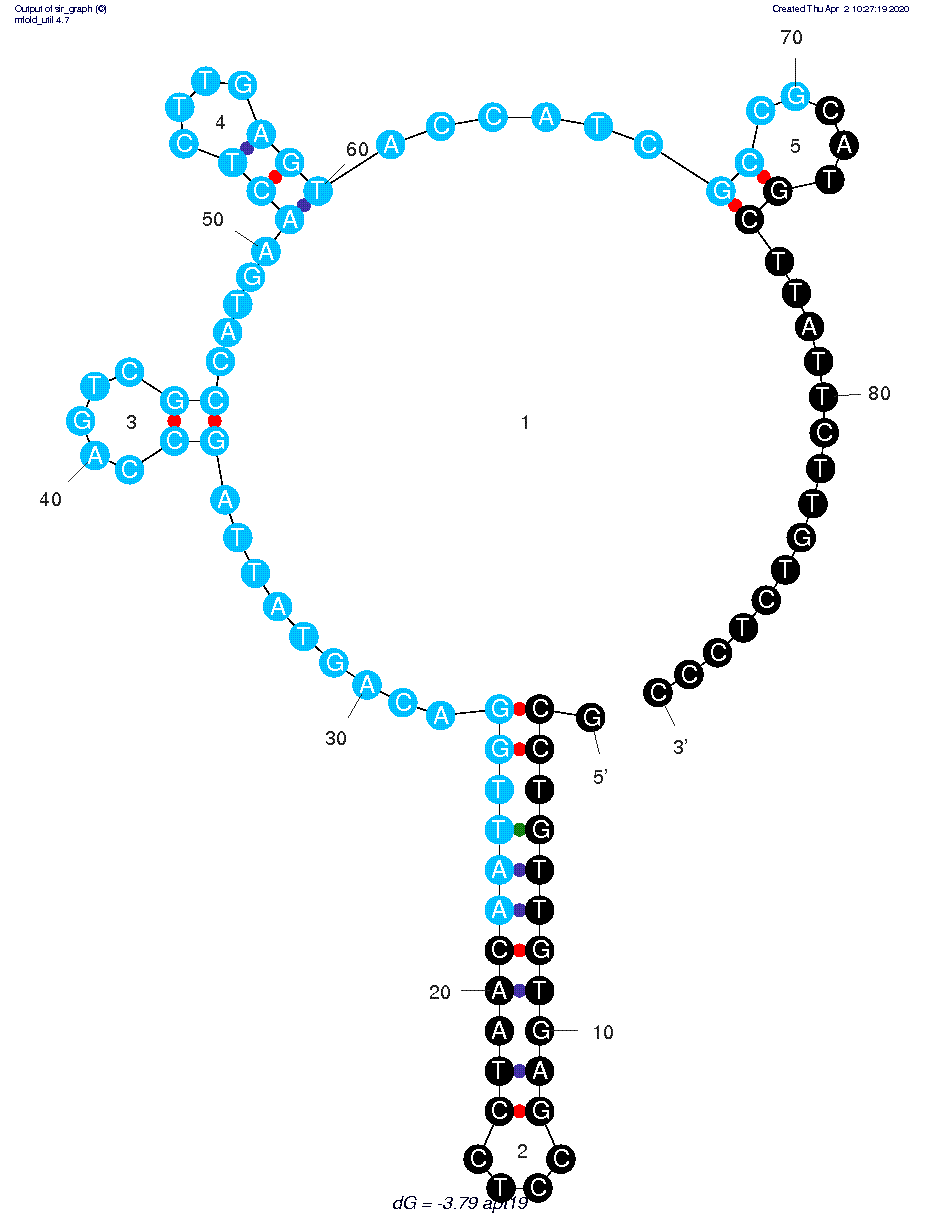  **Apt19, ∆G = -3.8** | 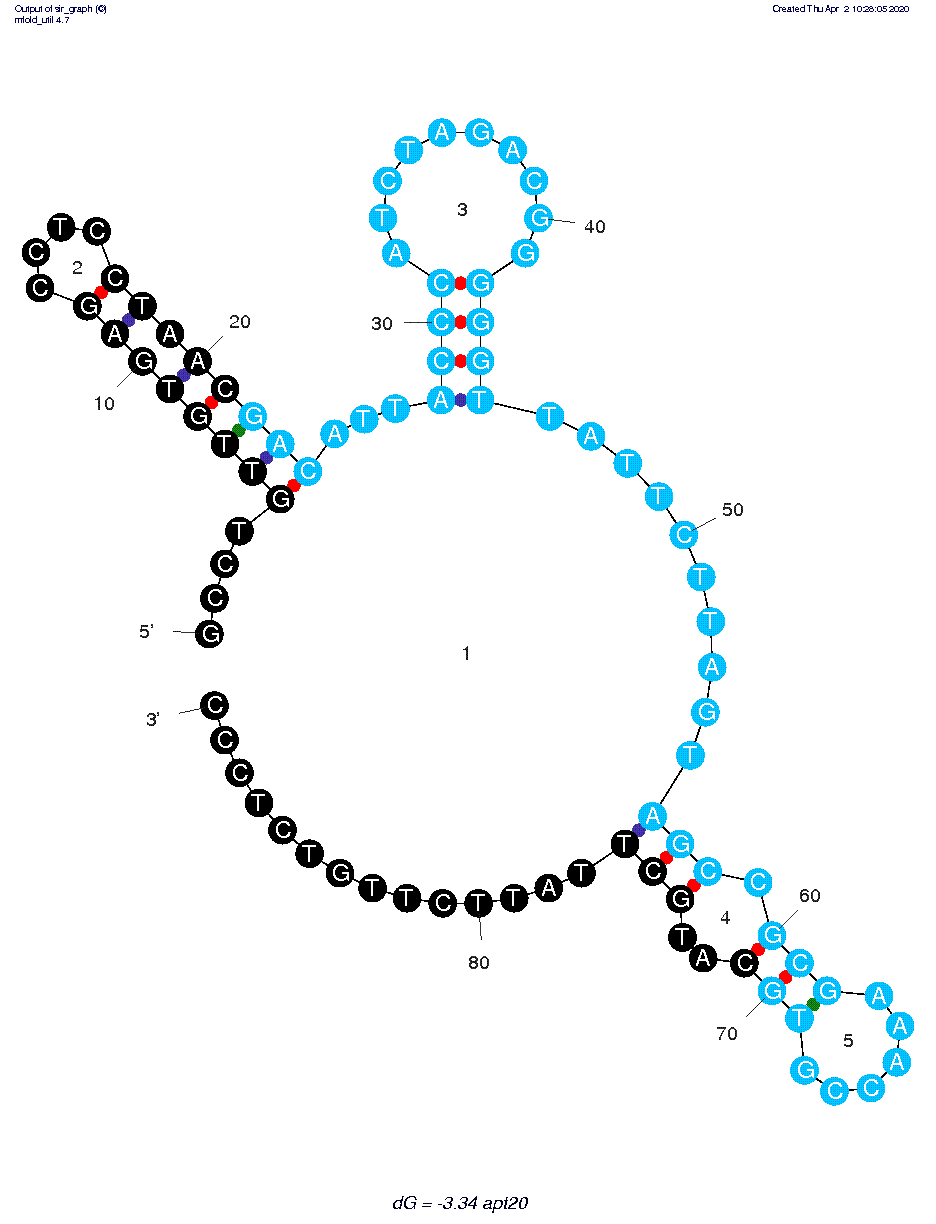  **Apt20, ∆G = -3.3** |
| 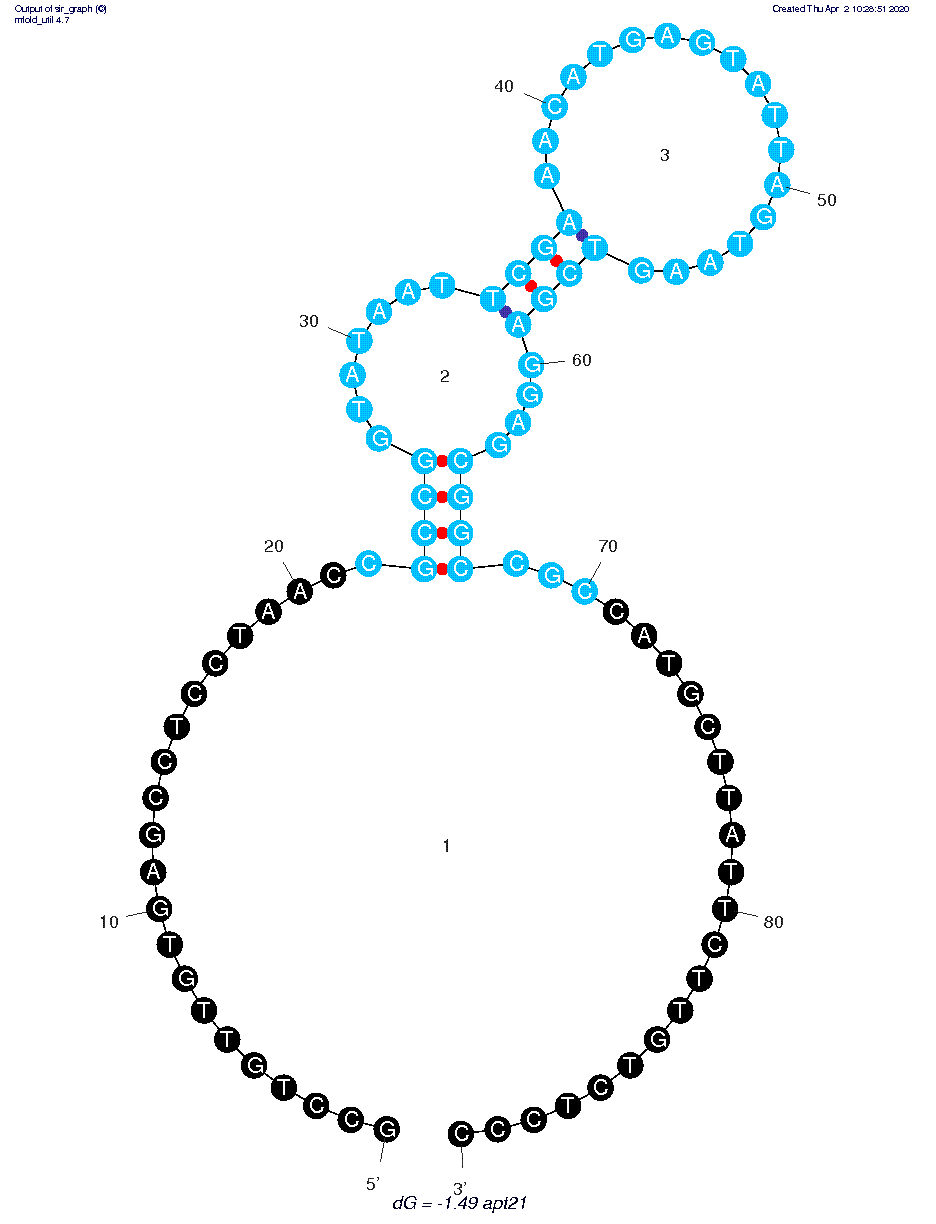  **Apt21, ∆G = -1.5** | 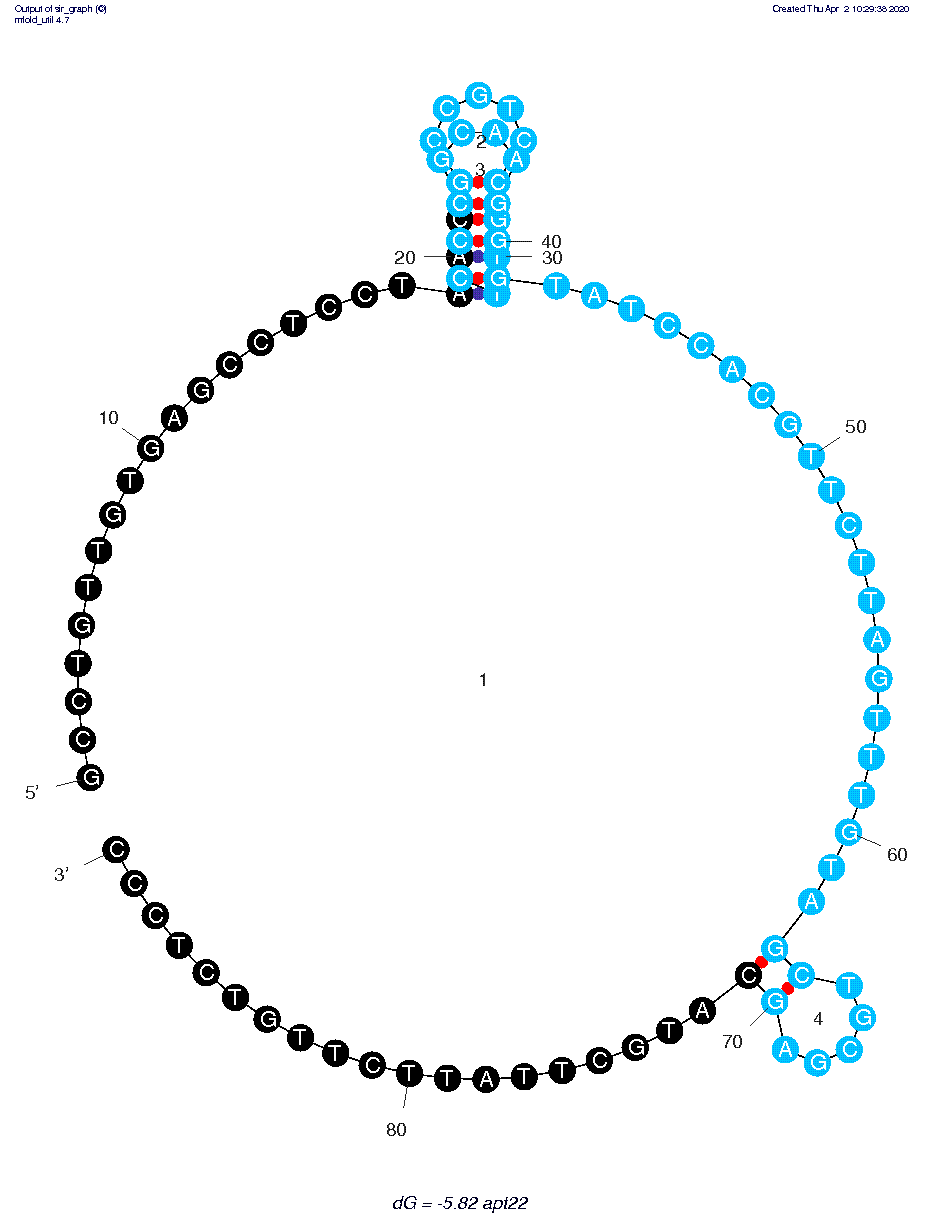  **Apt22, ∆G = -5.8** | 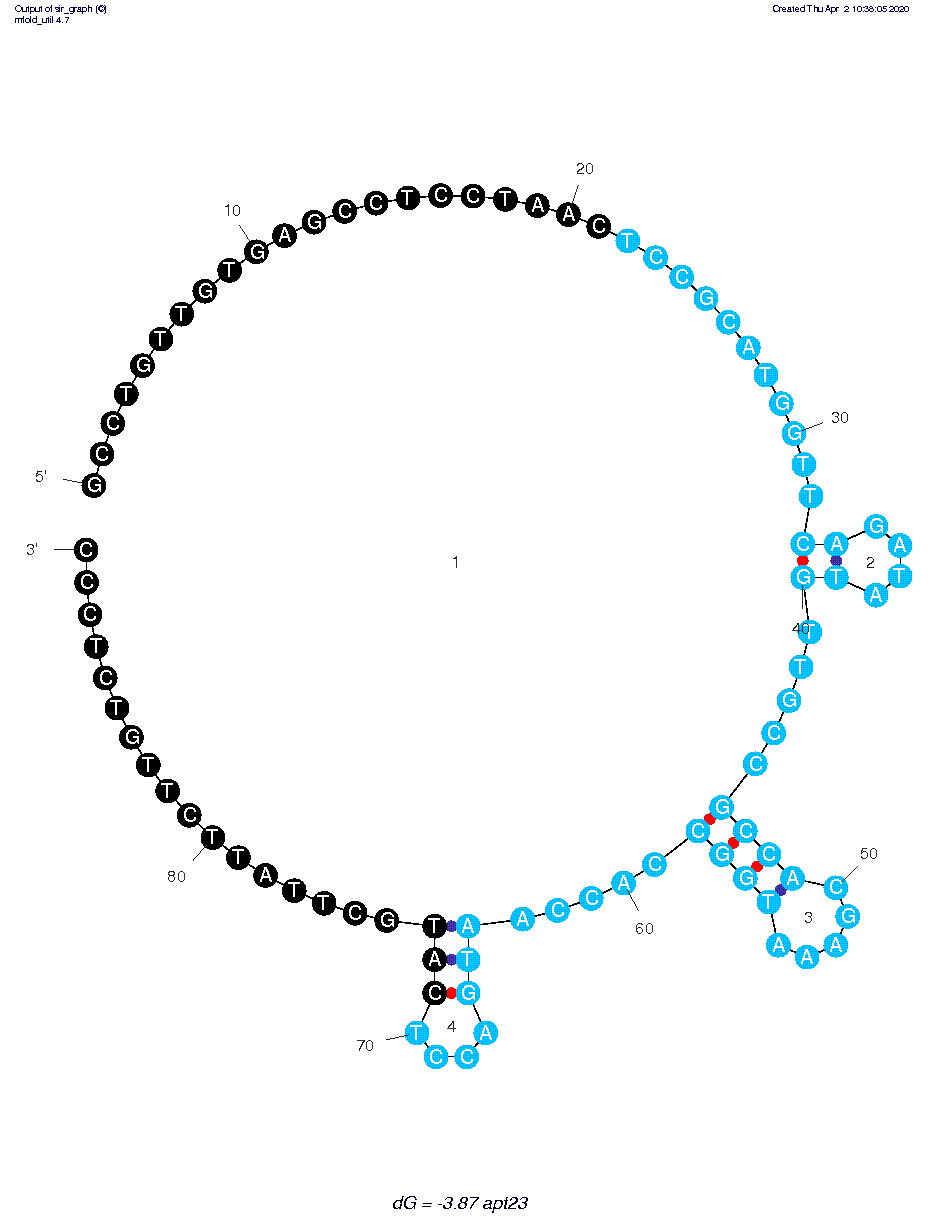  **Apt23, ∆G = -3.9** |
| 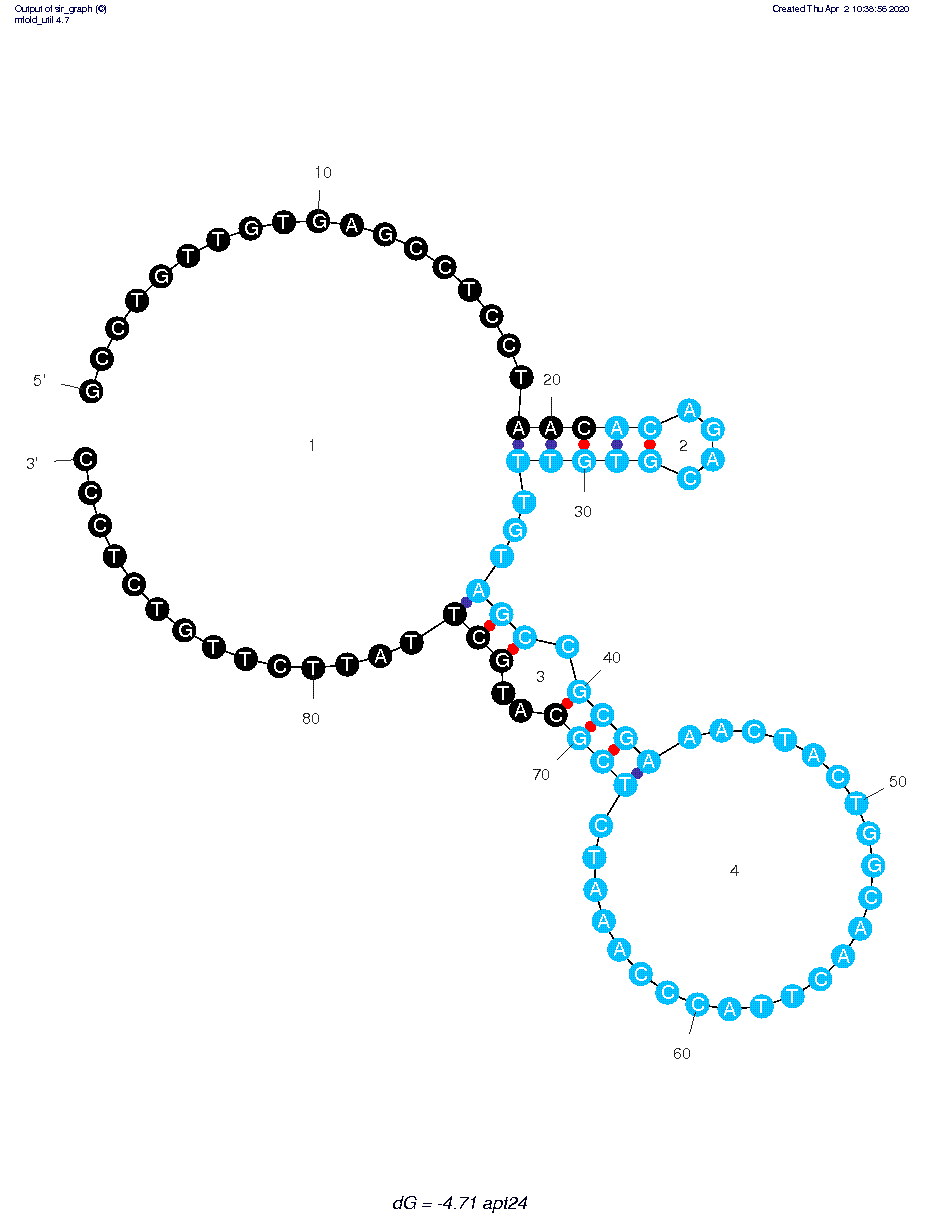  **Apt24, ∆G = -4.7** | 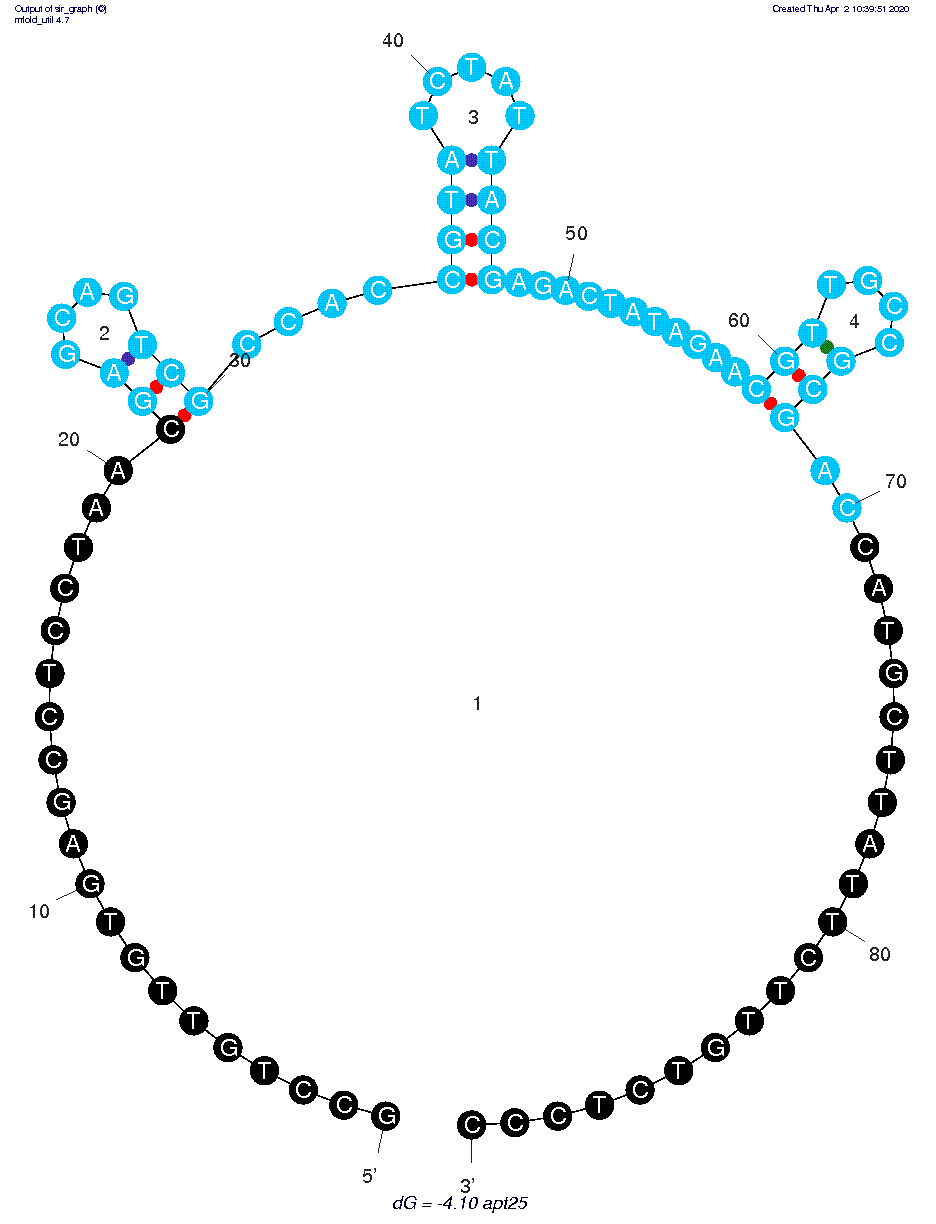  **Apt25, ∆G = -4.1** |  |
