# Supplementary material for: Selection of aptamers against triple negative breast cancer cells using high throughput sequencing
Source: Sci Rep. 2021 Apr 21;11:8614. doi: 10.1038/s41598-021-87998-y (PMC8060331; doi:10.1038/s41598-021-87998-y)
Supplement: Supplementary file 4 — Supplementary Information 4. [file 41598_2021_87998_MOESM4_ESM.docx]

**Supplementary Figure legends**

**Figure S1: Co-localization of Apt1 and Apt2 with MDA-MB-231 cells.** FAM-labelled Apt1 and Apt 2 were incubated at 37ºC with MDA-MB-231 cells and then with CF 568 phalloidin. Fluorescence imaging of MDA-MB-231 cells stained with the high affinity F-actin probe CF 568 phalloidin (1), incubated with FAM-labelled (2) Apt1 (a) and Apt2 (b).and stained with DAPI nucleic-acid specific (3). Merged files are representative of co-localization of Apt1 and Apt2 with phalloidin (4). All pictures were taken under fluorescence microscope using a 60X oil immersion objective. White arrows point membrane staining overlay with aptamer binding.

**Figure S2:** **Binding specificity of Apt1 and Apt2 aptamers to other breast cancer cell lines.** A) FAM-labelled Apt1 and Apt2 were incubated at 37 °C with MCF-7 and MDA-MB-453 cell lines and analyzed by flow cytometry. Two-way ANOVA indicates statistically significant differences within the group assessed by Sidak’s post-test and denoted as follows: **** ρ ≤ 0.0001. B) Microscopy results showing the fluorescence imaging of MCF-7 (a and b) and MDA-MB-453 (c and d) cells incubated with FAM-labelled Apt1 and Apt2 at 37 °C, respectively. (1) blue filter, nuclei stained with DAPI, (2) green filter, Apt1 or Apt2, and (3) merge of all filters (1 and 2).
